# Comparing and Optimizing Tissue Clearing Protocols for Cochlear Immunostaining and 3D Reconstruction by Light-Sheet Microscopy

**DOI:** 10.1007/s10162-026-01068-6

**Published:** 2026-08-24

**Authors:** Franziska M. K. Becker, Gina Dunkel, Martina Giampetraglia, Bettina Weigelin, Ellen Reisinger

**Affiliations:** 1 Gene Therapy for Hearing Impairment Group, Department for Otolaryngology – Head & Neck Surgery, University Hospital and Faculty of Medicine, University of Tübingen, Tübingen, Germany; 2 Werner Siemens Imaging Center and Department for Preclinical Imaging and Radiopharmacy, University Hospital and Faculty of Medicine, University of Tübingen, Tübingen, Germany; 3 Cluster of Excellence iFIT (EXC 2180) “Image-Guided and Functionally Instructed Tumor Therapies”, University of Tübingen, Tübingen, Germany

**Keywords:** Tissue clearing, Cochlear imaging, Ethyl cinnamate, iDISCO, cDISCO, PEGASOS, Sca*l*eS, FRUIT, Hair cells, Spiral ganglion neurons

## Abstract

**Purpose:**

Current standard methods for immunohistochemical analysis of the inner ear rely on cryosectioning of the intact cochlea or dissection of the organ of Corti. Cryosections provide only limited structural information and do not allow quantification of hair cell numbers per basilar membrane length, whereas dissections require substantial technical expertise and often result in damage to the basal cochlear turns. Tissue clearing has largely overcome these limitations and has been widely applied to organs such as the brain, heart, liver, and kidney. However, despite several reports describing cochlear clearing, a systematic comparison of diverse methods is lacking.

**Methods:**

Here, we present a comparative evaluation of five established clearing protocols applied to the mouse cochlea, including three organic solvent-based (applied to *n* = 10 cochleae each) and two aqueous-based approaches (*n* = 4/8).

**Results:**

Among these, iDISCO/cDISCO (i/cDISCO) and ethyl cinnamate (ECi) provided superior tissue transparency, morphological preservation, and immunofluorescence staining quality. In contrast, PEGASOS resulted in reduced transparency and occasional wave-like distortion of hair cell rows, probably due to uneven tissue shrinkage. Sca*l*eS and FRUIT exhibited limited clearing performance and variable staining results. Based on these findings, we optimized the two best-performing protocols to enable cochlear clearing within only five days, using inexpensive and minimally toxic reagents.

**Conclusion:**

The resulting fastDISCO and fastECi protocols achieved clearing and immunolabeling quality comparable to their full-length counterparts while preserving tissue morphology (applied to *n* = 17/20 cochleae). These rapid clearing protocols support reproducible 3D light-sheet microscopy of immunostained cochleae and enable automated cell segmentation and quantitative analysis of distinct cell types, including the inner and outer hair cells.

**Supplementary Information:**

The online version contains supplementary material available athttps://doi.org/10.1007/s10162-026-01068-6.

## Introduction

Traditionally, analyzing the structure of the sensory tissue of the inner ear, with its characteristic helical shape, has relied on whole-mount preparations of the organ of Corti or thin, typically mid-modiolar, sections of the cochlea [1]. Both approaches involve physical sectioning of the tissue, which inevitably leads to loss of structural information [2, 3]. Preserving the three-dimensional architecture of the cochlea is particularly important for accurately quantifying hair cells in the basal region, where most forms of progressive age-related and noise-induced hearing loss first manifest. However, an intact cochlea cannot be examined directly under a light microscope. Biological tissues, including the cochlea, are composed of diverse components such as extracellular matrix proteins, calcified bones, lipids, proteins, and nucleic acids, each of which has a distinct refractive index (RI), which causes light scattering [2, 4–6]. Moreover, endogenous pigments such as heme, lipofuscin, and melanin substantially color organs and blood vessels. Together, light scattering and absorption make it impossible to image untreated thick volumetric samples with light microscopy (reviewed by [2]).

Tissue clearing overcomes these limitations by homogenizing the RI within the sample and by reducing light absorption of endogenous pigments, thereby reducing scattering and rendering the tissue transparent [2, 7]. Originally developed by Werner Spalteholz in the early twentieth century, tissue clearing has received much attention in recent years, resulting in the refinement of implemented protocols and the development of new clearing methods [6, 8]. Tissue clearing techniques are now used to clear various organs, ranging from soft tissues like brain, lung and liver to calcified bone and dental tissues as well as from small to big samples such as organoids to intact mice [9–12]. Clearing methods are generally categorized into three types based on the chemical nature of the clearing solution: hydrophobic, hydrophilic, and hydrogel-based. Despite methodological variations, these protocols typically share certain key steps: fixation, permeabilization, delipidation, decolorization, dehydration, and RI matching [13].

Although hydrogel-based protocols can achieve excellent clearing capabilities, they require a specialized and costly equipment, including special perfusion procedures, expensive equipment for degassing the sample prior to the polymerization step, and special electrophoresis chambers [7, 14]. Therefore, in the present study, we focus on hydrophobic and hydrophilic approaches, which can be implemented using standard laboratory equipment [2, 5, 7].

Hydrophobic clearing methods employ organic solutions for RI matching. The most common reagents used are methyl salicylate, benzyl alcohol benzyl benzoate (BABB), ethyl cinnamate (ECi), and dibenzyl ether (DBE). For the clearing solution to be able to penetrate the tissue, a dehydration step is necessary, for which alcohols like methanol, ethanol, tert-butanol, or the ether tetrahydrofuran (THF) are employed [2, 5, 6]. While organic solvent–based methods generally yield excellent clearing results within short time frames, their reagents are often toxic or carcinogenic and can induce tissue shrinkage or deformation due to the dehydration [2, 4, 7]. In contrast, hydrophilic-based methods use mostly non-toxic agents — typically glycerol or concentrated sugar solutions — for RI matching, sometimes supplemented with chaotropic agents such as urea to enhance tissue permeability [7]. While these methods are less toxic and environmentally friendly, they require long incubation times, involve viscous solutions that are difficult to handle, and were reported to cause tissue swelling [2, 5].

Several clearing protocols were previously applied to the mouse cochlea. The hydrophobic-based method iDISCO was developed by Renier et al. [15] and uses DBE as RI matching solution. In 2021, it was adapted to cochlear clearing, hence called cDISCO [16]. Keppeler et al. [﻿^16^] cleared mouse cochleae as well as a marmoset temporal bone successfully and tested five methods, iDISCO, cDISCO, TDE, SeeDB, and Sca*l*eS, but reported only the results from cDISCO which they found superior over the others. Klingberg et al. [4] introduced ethyl-3-phenylprop-2-enoate (ethyl cinnamate, ECi), as a non-toxic clearing reagent. It efficiently cleared different internal organs and the paw of a mouse, and was shortly thereafter adapted to use in cochlear clearing [17, 18]. At about the same time, a hydrophilic-based protocol, Sca*l*eS [19] was adapted for the cochlea, and reportedly outperformed 3DISCO, iDISCO, CLARITY, and CUBIC in terms of tissue transparency [20].

Here, we systematically compared seven clearing strategies for their ability to clear the mouse cochlea and homogeneously immunolabel hair cells and spiral ganglion neurons (SGNs), while preserving the morphology of the cochlea. Three of these protocols were previously applied to the cochlea, i/cDISCO, ECi, and Sca*l*eS, and two were adapted for mouse cochleae for the first time, PEGASOS and FRUIT [9, 10, 21]. We further developed and evaluated two shortened variants of i/cDISCO and ECi, which enable efficient clearing and immunostaining of the mouse cochlea within only 5 to 7 days.

## Methods and Materials

### Animals

Animal care, procedure, and experimental protocols correspond to national and institutional guidelines and were reviewed and approved by the Veterinary Care Unit of the University of Tübingen and the Animal Care and Ethics Committee of the regional board of the Federal State Government of Baden-Württemberg, Germany. All experiments were performed according to the European Union Directive 2010/63/EU for the protection of animals used for experimental and other scientific purposes. In-house bred mice were kept according to the national guidelines for animal care in a specific pathogen-free animal facility at 25 °C on a 12 h/12 h-light/dark cycle, with average noise levels of around 50–60 dB sound pressure level. Young (P28-P36) as well as adult (2–9 months) mice of both sexes with C57BL/6N background (Charles River, France) were used within this study. Mice were anesthetized with slow infusion of CO_2_ (30% cage volume per min) before decapitation or with ketamine/xylazine i.p. before transcardial perfusion.

### Tissue Processing

#### Cochlea Preparation and Fixation

The inner ears were separated from the scull and collected in ice cold phosphate-buffered saline (PBS). The cochleae were extracted under visual control using a stereomicroscope, and connective tissue was removed while semicircular canals remained. A small hole was made at the apical part of the cochlea, near the helicotrema. The oval as well as the round window were gently pierced with forceps to perforate the membrane (Supplementary Fig. S1 a), to allow solutions to enter by passive diffusion. Fixation was performed with 4% formaldehyde (FA) in PBS (see below for duration and incubation temperature).

#### Permeabilization and Antibody Incubation

Samples were permeabilized and unspecific antibody binding sites were blocked by incubation in donkey-serum based blocking buffer (DSDB, 17% donkey serum, 0.3% Triton X-100, 20 mM phosphate buffer, 0.45 M NaCl) for the specified duration. Antibodies were diluted in DSDB and incubated at 37 °C unless otherwise indicated. All steps involving fluorescently labelled secondary antibodies were performed under light protection. No rocking or shaking was used at any time, since we otherwise observed tissue rupture. Primary antibodies included rabbit anti-myosin VIIa (Proteus Biosciences Inc., 25–6790, 1:400), rabbit anti-oncomodulin (Swant, OMG4, 1:200), rabbit anti-beta III tubulin (Sigma-Aldrich, T2200, 1:100), goat anti-calretinin (Swant, CG1, 1:200), guinea pig anti-parvalbumin (Synaptic Systems, 195004, 1:450), and guinea pig anti-Vglut3 (Synaptic Systems, 135204, 1:400). Secondary antibodies included Alexa Fluor™ 546 donkey anti-rabbit IgG H + L (Invitrogen, A10040, 1:200), Alexa Fluor™ 594 donkey anti-goat IgG H + L (Invitrogen, A11058, 1:400), Alexa Fluor® 647 donkey anti-rabbit IgG H + L (Jackson ImmunoResearch, 711–606-152, 1:800), Alexa Fluor™ Plus 647 donkey anti-goat IgG H + L (Invitrogen, A32849, 1:400), and Alexa Fluor® 790 donkey anti-guinea pig IgG H + L (Jackson ImmunoResearch, 706-655-148, 1:400). As nucleic marker SYTOX™ Green nucleic acid stain (Invitrogen, S7020, 1:5000) was used. For further details, please see Supplementary Table S1.

#### Tissue Trimming and Agarose Embedding

Semicircular canals, connective tissue, blood vessels, and parts of the bone were removed from the cochlea to further minimize light adsorption and scattering (Supplementary Fig. S1 b + c). Trimmed cochleae were embedded in low melt agarose to ease fixation to the microscope holder and to ensure precise positioning of the cochlea, such that the modiolar axis was perpendicular to the optical pathway. For embedding, we cut a 1-mL Insulin syringe to the edge of the start of the plunger (Supplementary Fig. S1 d). The plunger was drawn up, 70 µL hot 1% low-melt agarose (w/v in ddH_2_O, Carl Roth, 6351.1) was pipetted into the top of the syringe, and the cochlea was carefully inserted into the agarose. Following cooling on ice, the agarose block containing the cochlea was transferred back to a reaction tube to proceed with the clearing protocol (Supplementary Fig. S1 e).

### Clearing Protocols

We compared the organic solvent-based clearing protocols ECi [17], i/cDISCO (based on iDISCO [15], and cDISCO [16]) and PEGASOS [9] as well as the water-based clearing protocols Sca*l*eS [20] and FRUIT [21] using modified incubation times to adapt to the small size of the mouse cochlea. See Supplementary Fig. S1 f for a schematic illustration of the different steps and lengths of original protocols.

### Organic Solvent-Based Tissue Clearing Protocols

#### Original Length Ethyl Cinnamate (ECi) Protocol

We adjusted the protocol from Brody et al. [17] to the mouse cochlea as follows: cochleae were harvested as described above and fixed in PBS containing 4% formaldehyde (FA) at 4 °C overnight. After washing three times for 10 min with PBS at RT, samples were decalcified in 200 mM EDTA (Tetrasodium Tetrahydrate Salt, MERCK, #34103-500GM, in ddH_2_O) at RT for 2.5 weeks, with the solution being exchanged every 2 days. Next, cochleae were incubated in DMSO with three changes every 10 min and washed with PBS three times 20 min each. The samples were then blocked and permeabilized with DSDB for 1 h at RT, primary antibodies in DSDB were incubated for three days and secondary antibodies overnight at 37 °C, with washing with PBS-T (1% Triton-X100) at 37 °C at least three times in between. After secondary antibody binding, samples were washed three times with PBS-T at 37 °C and twice with PBS at 4 °C. The cochleae were embedded in 1% low-melt agarose as described above (Supplementary Fig. S1 b-e). For dehydration, the cochleae were placed in a series of increasing concentrations of ethanol/ddH_2_O (pH > 9.0; 50%, 70%, 100%, 100%) for up to 12 h each at 4 °C and finally submerged in ECi overnight at RT. The approximate duration of this protocol is 26 days.

#### FastECi

Compared to the original ECi protocol, we modified the fixation and decalcification process, refined the dissection of the cochleae, and shortened all incubation steps.

Cochleae were harvested as described, with an extra trim of the bony shelf behind the apical tip of the cochlea (see Fig. 2a + b), before opening a hole in the bony shelf at the cochlear apex and gentle puncturing the oval and round window membranes. Samples were fixed in PBS containing 4% FA for 3 h at 4 °C. The samples were washed 3 × 10 min in PBS followed by partial decalcification at 37 °C in 200 mM EDTA for 30 to 180 min (depending on the age of the animal, see Supplementary Table S2), before further dissection took place: The bony shelf at the back of the cochlea (optional), semicircular canals, and remaining blood vessels were removed (Fig. 2c–f). Cochleae were then fully decalcified at 37 °C for 24 h (or 3 days when the bony shelf at the back of the cochlea remained). The samples were rinsed 3 × with DMSO for 10 min and 3 × with PBS for 20 min. For permeabilization, cochleae were incubated in DSDB for 3 h at 37 °C. For immunohistochemistry, primary antibodies were incubated overnight at 37 °C. Cochleae were rinsed 3 × 10 min with PBS-T (1% Triton-X100) at 37 °C. Secondary antibodies were incubated likewise. Samples were rinsed with PBS-T 3 × for 15 min at 37 °C followed by 2 × 15 min in PBS at 4 °C. During the last washing step, any remaining connective tissue and visible blood vessels were removed. The cochleae were embedded in 1% low-melt agarose followed by dehydration with a serial dilution of ethanol/ddH_2_O, i.e., 20%, 40%, 60%, 80% and 100% ethanol for 1 h each at RT. Homogeneous dehydration was ensured by inverting the tubes occasionally. Finally, samples were incubated in fresh 100% ethanol overnight at RT, followed by submersion in ECi for at least one night at RT. The approximate duration of this protocol is 5 days (or 7 days with less trimming).

#### Original Length i/cDISCO

The original length i/cDISCO protocol, based on the iDISCO protocol from Renier et al. [15] and the cDISCO protocol according to Keppeler et al. [16], was performed as follows: Cochleae were harvested as described above and fixed in PBS containing 4% FA at 4 °C overnight, followed by 3 days of decalcification in 200 mM EDTA at RT. After decalcification, a 2-day decolorization step with 25% N,N,N’,N’-Tetrakis(2-Hydroxypropyl)ethylenediamine (Quadrol) was performed, followed by 2 × 1 h incubation in PTx.2 (PBS/0.2% Triton X-100) at RT and incubation in PTD (PBS/0.2% Triton X-100/20% DMSO) at 37 °C overnight, according to the “alternative methanol-free pretreatment” by Renier et al. [15]. Samples were then incubated with PTTDDI (PBS/0.1% Tween-20/0.1% Triton X-100/0.1% Deoxycholate/0.1% IGEPAL Ca-630/20% DMSO) at 37 °C overnight and again washed 2 × 1 h in PTx.2 at RT. For immunostaining, samples were incubated for 2 days in permeabilization solution (2.3% Glycine/20% DMSO/ad PTx.2) at 37 °C and 2 days in blocking solution (DSDB) at 37 °C. Primary antibodies were diluted in DSDB and incubated at 37 °C for 14 days. Samples were washed 4–5 times in PTwH (0.2% Tween-20/0.1% 10 mg/ml Heparin Stock/10% 10 × PBS/ad H_2_O) until the next day. Secondary antibodies were diluted in DSDB and incubated for 14 days at 37 °C. Again, samples were washed 4–5 times in PTwH until the next day. After immunostaining, cochleae were embedded in 1% low-melt agarose (Supplementary Fig. S1 b-e). For dehydration, a methanol/ddH_2_O series of 20%, 40%, 60%, 80%, and 100% each 1 h at RT was employed. Samples were left in fresh 100% methanol overnight, followed by 66% dichlormethane (DCM)/33% methanol incubation for lipid extraction for 3 h at RT. Samples were then washed twice with 100% DCM for 15 min. Lastly, samples were immersed in Dibenzylether (DBE) for at least 24 h. The approximate duration of this protocol is 44 days.

#### FastDISCO

Compared to the long i/cDISCO protocol, we modified the fixation and decalcification process, refined the dissection of the cochleae, and shortened all incubation steps.

Cochleae were harvested, and the bony shelf behind the apical tip of the cochlea was trimmed as described for fastECi (Fig. 2a + b). The cochleae with a hole at the apex and pierced oval/round window membranes were fixed in PBS containing 4% FA at 4 °C for 3 h. The samples were washed 3 × 10 min in PBS followed by partial decalcification at 37 °C in 200 mM EDTA for 30 to 180 min (depending on the age of the animal, Supplementary Table S2), before the bony shelf at the back of the cochlea (optional), semicircular canals and remaining blood vessels were removed (Fig. 2c–f). Cochleae were then fully decalcified for 24 h at 37 °C (or 3 days when cochlear bone was not removed), washed 3 × 10 min with PBS and decolorized with 25% Quadrol solution for 2 h. The methanol-free pretreatment was shortened in incubation time: Samples were washed 3 × 10 min in PTx.2, 3 × 10 min at 37 °C in PTD, 3 × 10 min at 37 °C in PTTDDI and lastly 3 × 10 min in PTx.2. Cochleae were then incubated for 30 min at 37 °C in permeabilization solution followed by 2 h in DSDB at 37 °C. Primary antibodies were applied in DSDB as described and incubated overnight at 37 °C. Samples were washed 3 × 10 min with PTwH at 37 °C and secondary antibodies were incubated likewise. Samples were again washed 3 × 10 min with PTwH at 37 °C. If needed, any remaining connective tissue and macroscopic blood vessels were removed before embedding the samples in 1% low-melt agarose. Methanol was replaced by ethanol in all steps. Dehydration was performed in an ethanol/ddH_2_O series of 20%, 40%, 60%, 80% and 100%, each 1 h at RT, with leaving the cochlea in fresh 100% ethanol for overnight incubation at RT. Homogeneous dehydration was ensured by inverting the tubes occasionally. DCM incubation was performed with 66% DCM/33% ethanol for 3 h at RT, followed by 100% DCM twice for 15 min. Clearing was performed in DBE overnight. The approximate duration of this protocol is 5 days (or 7 days for the less trimmed cochlea).

#### PEGASOS

We adapted the PEGASOS protocol described by Jing et al. [9] as follows: Cochleae were harvested as described above and fixed in PBS containing 4% FA at 4 °C overnight, followed by four days of decalcification in 200 mM EDTA at 37 °C. After decalcification, decolorization was performed with 25% Quadrol for two days and 5% ammonium solution (ammonia solution 25%, Merck, 1.05432.1011) for 6 h, each incubated at 37 °C. For immunohistochemistry, samples were blocked with DSDB and incubated with primary antibody in DSDB for 3 days at 4 °C. The samples were washed overnight in PBS at RT and incubated with secondary antibodies in DSDB for three days at 4 °C. After secondary antibody incubation, the samples were washed in PBS for 6 h. Cochleae were embedded in 1% low-melt agarose as described above (Supplementary Fig. S1 d + e) and incubated in PBS overnight. Samples were placed in a gradient of tert-butanol/H_2_O solutions (30% for 4 h, 50% for 6 h, 70% for 24 h) for delipidation at 37 °C. Dehydration was done using 70% tert-butanol/30% PEG-MMA-500 solution for two days at 37 °C, with daily exchange of the solution. For RI matching, samples were placed in a solution of 75% benzyl benzoate/25% PEG-MMA-500 and incubated for one day at 37 °C. Delipidation, dehydration, and clearing solutions all contained 3% of Quadrol each. The approximate duration of this protocol is 20 days.

### Water-Based Tissue Clearing Protocols

#### Sca*l*eS

Clearing using the Sca*l*eS protocol was performed according to the protocol by Urata et al. [20] with minor modifications. Cochleae were harvested as described above and fixed in PBS containing 4% FA at 4 °C overnight, followed by 5 days of decalcification in 200 mM EDTA at 37 °C. After several times of washing with PBS, samples were incubated in GuSGluT solution (3 M guanidinium chloride/35% (w/v) D-sorbitol/15% (w/v) D-glucose/4% (w/v) Triton X-100 in PBS; pH 6.0–8.0) at 37 °C for 6 h. Samples were washed in PBS-T (0.1% Triton-X100) for 30 min at RT, blocked in DSDB at 37 °C overnight and then incubated with primary antibodies in DSDB for 48 h at 37 °C. Next, samples were washed for 30 min with PBS-T at RT. Incubation with secondary antibodies in DSDB was performed for 48 h at 37 °C, followed by another 30 min washing step in PBS-T at RT. Cochleae were embedded in 1% low-melt agarose (Supplementary Fig. S1 b-e) and subsequently incubated for 2 h at 37 °C in RI-matching solution (3 M guanidinium chloride/60% (w/v) D-sorbitol/0.1% (w/v) Triton X-100 in PBS; pH 7.1). The approximate duration of this protocol is 13 days.

#### FRUIT

Clearing was performed according to the optimized FRUIT protocol by Hou et al. [21]. Cochleae were harvested, fixed in PBS containing 4% FA at 4 °C overnight, and decalcified in 200 mM EDTA for 2 days at 37 °C. After 3 × 10 min washing in PBS at RT and blocking in DSDB at 37 °C overnight, antibodies diluted in DSDB were incubated at 37 °C for 3 days each, with washing 3 × 10 min with PBS in between and after secondary antibody incubation. Cochleae were embedded in 1% low-melt agarose (Supplementary Fig. S1 b–e), and then incubated in a series of FRUIT dilutions (w/v): 8 h in 35%, overnight in 40%, 8 h in 60%, overnight in 80% and 24 h in 100% at RT. The FRUIT solutions contained 0.5% α-thioglycerol with increasing concentrations of fructose and decreasing concentrations of urea (e.g., 35% FRUIT contained 35% (w/v) fructose and 8 M urea, see [21]). Samples were stored in 100% FRUIT at 4 °C. The approximate duration of this protocol is 13 days.

### Light-Sheet Microscopy

Cleared cochleae were imaged using a light-sheet fluorescence microscope (Ultramicroscope II Blaze, Miltenyi Biotec). Samples embedded in low-melt agarose were mounted on the imaging holders using super glue (Drechseln und Mehr, CA-Kleber Maxi-Cure™ dickflüssig #4115603; InstaSet Accelerator #4119902) and submerged in refractive index matching solution in the imaging chamber. DBE-cleared samples were transferred to ECi for imaging. Before mounting, samples were incubated in ECi for at least 1 h and then placed into the imaging chamber containing fresh ECi. ECi was used as the imaging medium for all organic solvent-cleared samples to keep imaging conditions consistent and to avoid repeated exchange of the microscope chamber solution. Water-based cleared samples were imaged in the respective clearing solution of Sca*l*eS or FRUIT. Refractive-index differences between aqueous and organic clearing solutions were accounted for optically during image acquisition by using the appropriate exchangeable dipping cap for the MI Plan objective of the UltraMicroscope Blaze system. All datasets were acquired using a 12 × objective with 1.0 × magnification as mosaic scans with 15% overlap between tiles. Entire cochleae were imaged as volumetric stacks, covering the full apical-to-basal extent of the organ of Corti. Z-stacks were acquired with a constant step size of 2 µm. Samples were illuminated from both sides to improve signal uniformity. Fluorescence excitation (ex.) and emission (em.) channels were selected according to the fluorophore-conjugated secondary antibodies used for immunostaining (SytoxGreen, ex.: 470/40, em.: 525/50; AlexaFluor594, ex.:560/40, em.: 620/60; AlexaFluor647, ex.: 630/20, em.: 720/60; DyLight755, ex.: 740/40, em.: 780lbp). All samples were imaged using identical acquisition settings to allow direct qualitative and semi-quantitative comparison between clearing protocols.

### Image Processing and Quantification

#### Assessment of Tissue Transparency

Tissue transparency after clearing was assessed using brightfield images of cleared cochleae. Images were acquired under identical illumination and exposure settings to allow comparison between different clearing protocols. Brightfield images were imported into Fiji/ImageJ for analysis.

To visualize residual light absorption and scattering across the sample, a straight line was manually drawn through the cochlea in the brightfield image. The line extended beyond the visible tissue boundaries to encompass the transition from the surrounding imaging medium to the tissue and back into the medium. The corresponding intensity profile was extracted as a transmitted-light profile and normalized to its own intensity range to display relative transmitted-light intensity. Lower values indicate reduced transparency due to residual absorption or scattering. Because sample orientation, residual pigmentation, illumination, and RI-matching conditions can influence absolute intensity values, the normalized line profiles were interpreted as semi-quantitative visualizations of relative transparency changes within each sample rather than as absolute measurements of transparency between protocols.

#### Evaluation of Immunofluorescence Staining Homogeneity

To assess antibody penetration and consequently immunostaining homogeneity along the organ of Corti, cleared cochleae were stitched using Imaris Stitcher (v10.1.0), imported into Imaris (Bitplane, v10.1.0) and rotated in three dimensions to obtain standardized top-view orientations. Top-view images were exported using identical display settings while avoiding pixel saturation. Fluorescence intensity along the hair cell row was quantified in Fiji/ImageJ by manually drawing a polyline from apex to base using a fixed line width of 15 pixels. The corresponding fluorescence intensity profile was extracted and normalized to the total length of the polyline (0 = apex, 1 = base). For visualization, normalized intensity profiles were color-coded and overlaid as proxy lines onto the corresponding top-view projections. Analyses focused on relative fluorescence intensity gradients along the organ of Corti rather than absolute fluorescence values, enabling comparison of staining continuity and signal preservation across different clearing protocols.

#### Automated Segmentation of Cochlear Hair Cells

Three-dimensional segmentation and quantitative analysis of cochlear hair cells were performed using Imaris software (version 10.1.0; Bitplane). Light-sheet microscopy datasets were imported without additional preprocessing beyond channel assignment. To enable layer-specific analysis, inner and outer hair cell regions were segmented separately. First, the approximate spatial extent of the inner and outer hair cell layers was delineated by generating two independent surface objects based on Vglut3 or oncomodulin immunofluorescence. These surfaces were used to define inner and outer hair cell regions of interest, respectively. Based on these surfaces, masked versions of the Vglut3 or oncomodulin channel were generated, isolating the signal corresponding to either the inner or the outer hair cell layer. Automated segmentation of individual hair cell nuclei was then performed independently for each layer using the Imaris machine learning-based classifier, applied to the Vglut3 or oncomodulin channel within the respective masked regions. Cells were segmented as individual objects without manual annotation.

## Results

### Comparing Established Organic and Water-Based Protocols for Clearing Cochleae

**Fig. 1:**
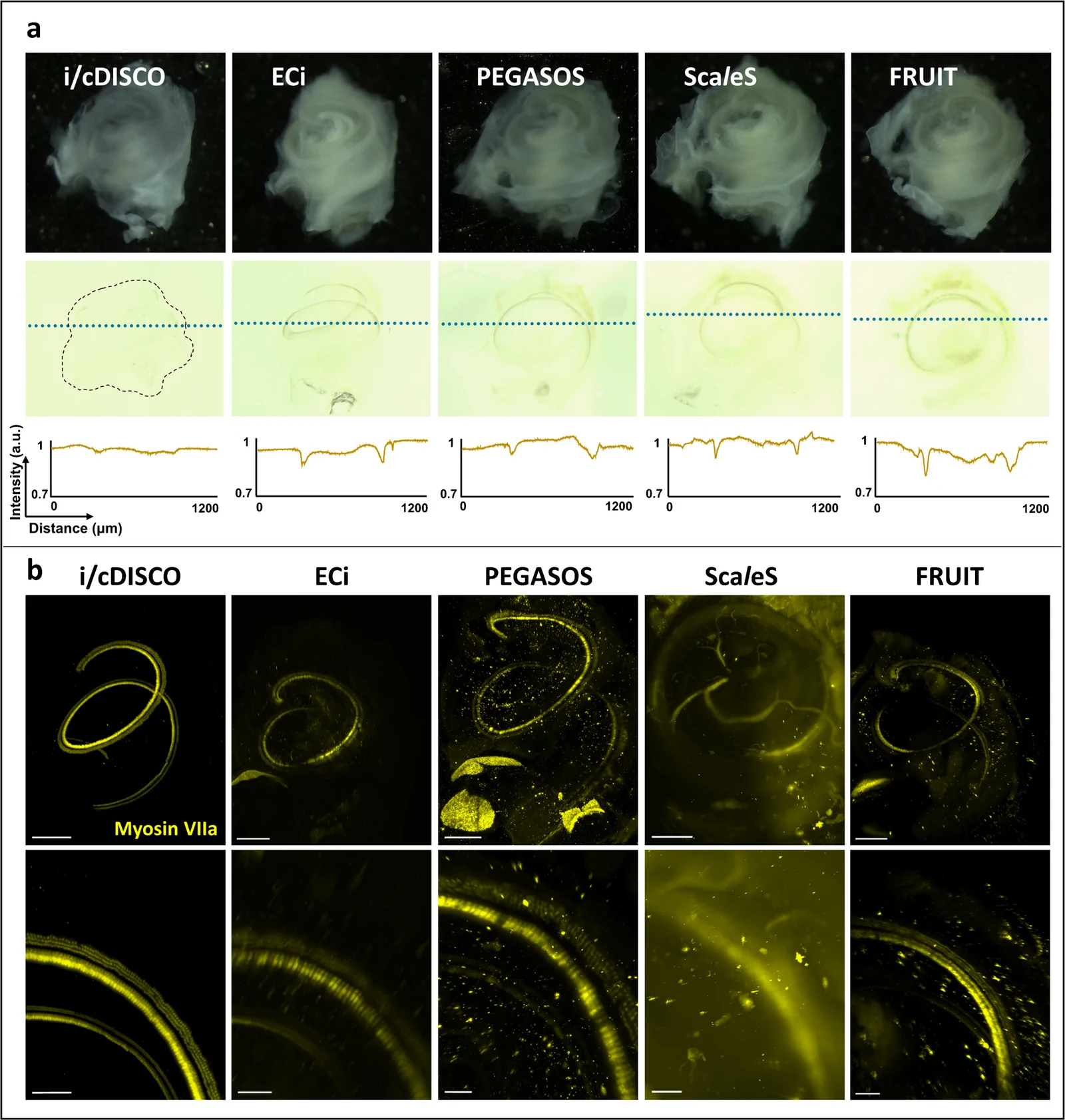
Comparison of cochlear transparency and immunofluorescence across different clearing methods. **a** Representative images of cochleae from 2–3 month old mice cleared with i/cDISCO (*n* = 10 independent experiments), ECi (*n* = 10), PEGASOS (*n* = 10), Sca*l*eS (*n* = 8), and FRUIT (*n* = 4). Top row: cochleae after EDTA incubation. Middle row: cochleae after clearing, imaged under a stereo microscope with top illumination. Bottom row: assessment of optical transparency using white cold light illumination. Representative transmitted-light line profiles are shown to visualize relative changes in transparency across each sample. Lower values indicate reduced transparency due to residual absorption or scattering. Blue dotted lines indicate where the profile was acquired. **b**, top row: 3D light-sheet microscopy reconstruction of cleared cochleae immunolabeled for myosin VIIa to visualize inner and outer hair cells; scale bar: 300 µm. Bottom row: Higher-magnification views of the apical region of the organ of Corti showing inner and outer hair cells; views were rotated for display to allow easier comparison across protocols. Scale bar: 100 µm

To compare established tissue clearing protocols for mouse cochleae, we selected three organic solvent-based (i/cDISCO, ECi, and PEGASOS [9, 15–17]) and two water-based protocols (Sca*l*eS and FRUIT [20, 21]). We evaluated each protocol for transparency, immunostaining continuity, signal-to-background quality, tissue morphology, and reliability (Fig. 1 and Table 1). Transparency was assessed visually with cold white light illumination in a stereo microscope based on the visibility of blood vessels, stria vascularis pigmentation, and residual bone (Fig. 1a, row 2 and graphs). For comparing immunostaining continuity and fluorescence intensity after clearing, we immunostained the cochleae for myosin VIIa, a well-characterized marker for inner and outer hair cells in the organ of Corti. The immunohistochemistry outcome was characterized as follows: Immunostaining continuity was determined by evaluating the presence of specific immunofluorescence as well as its homogeneity from apex to base in reconstituted 3D images (Fig. 1 and Supplementary Fig S4a). In addition, we qualitatively assessed the strength of the specific fluorescence signal in relation to unspecific background fluorescence. Morphological preservation was evaluated qualitatively based on visible tissue alterations. We also monitored the reproducibility of the clearing protocols by evaluating whether the best result was always or only occasionally achieved in these categories by independent experiments, and categorized this as the “reliability” of each protocol.

**Table 1:** Qualitative evaluation of five clearing protocols

	i/cDISCO	ECi	PEGASOS	Sca*l*eS	FRUIT
Transparency	4	3	2	1	1^2^
Immunostaining continuity	4	4	4	3	2
Signal-to-background quality	3^1^	3.5	2.5	2	2
Morphology	4	4	2.5	4	4
Reliability	2	4	2.5	1.5	0.5
Total (max: 20)	17	18.5	13.5	11.5	9.5

^1^When imaged within 1–2 weeks after clearing; longer incubation in DBE leads to increased autofluorescence in the stria vascularis

^2^FRUIT solution turns turbid within a few days, which further reduced transparency

Transparency was assessed based on residual visibility of bone, blood vessels and stria vascularis pigmentation in brightfield images. Immunostaining continuity was assessed based on the presence of specific immunofluorescence labelling along the organ of Corti from apex to base, considering major unlabelled regions or signal dropouts. Signal-to-background quality was qualitatively assessed based on the brightness of antibody labelling compared with nonspecific immunofluorescence or autofluorescence. Morphological preservation was evaluated based on visible distortion of the organ of Corti. Scores (max. 4 credits) summarize the overall evaluation of transparency, immunostaining continuity, signal-to background quality and morphology of those independent cochleae where some immunolabelling could be detected; reliability considers all independent experiments: i/cDISCO, *n* = 10; ECi, *n* = 10; PEGASOS, *n* = 10; Sca*l*eS, *n* = 8; FRUIT, *n* = 4

Of the organic solvent-based protocols, i/cDISCO resulted in outstanding transparency with almost invisible bone, stria vascularis and blood vessels (Fig. 1a, middle row). Prolonged incubation in DBE for >1 week removed the remaining pigments in the stria vascularis, rendering the cochlea completely transparent. In ECi-cleared cochleae, the bone appeared transparent, while pigments in stria vascularis and blood vessels remained visible (Fig. 1a, middle row). Among the organic solvent-based protocols, PEGASOS was least efficient in clearing the cochlea, leaving the bony shelf behind the apex still visible (Fig. 1a, middle row; Table 1).

To complement the visual assessment of cochlear transparency, we performed line profile-based analysis of brightfield images to visualize relative changes in transmitted light across the cleared tissue (Fig. 1a, bottom row). For each cochlea, grayscale intensity profiles were extracted along a straight line spanning the entire width of the sample from inverted brightfield images acquired under identical imaging conditions (Fig. 1a and Supplementary Fig. S3a). Line profiles confirmed that i/cDISCO-cleared cochleae exhibited relatively uniform transmitted light intensity across the selected tissue region, consistent with low residual absorption and scattering. By contrast, PEGASOS-, Sca*l*eS-, and FRUIT-cleared cochleae showed more pronounced local decreases in transmitted-light intensity along the selected line profiles, reflecting some residual light absorption by bone and pigmented structures.

All organic solvent-based protocols facilitated homogeneous staining and preserved immunofluorescence in a comparable manner, resulting in efficient labeling of all myosin VIIa-expressing cells in the organ of Corti (Fig. 1b). While ECi only occasionally resulted in incomplete antibody penetration, this occurred more frequently in i/cDISCO and PEGASOS, with near absence of specific labelling in some experiments. i/cDISCO and ECi led to the brightest and most homogeneous staining of hair cells from apex to base (Fig. 1b and Supplementary Fig. S4a). PEGASOS cleared samples displayed highly variable immunostaining signals along the cochlear axis over the course of several experimental repetitions. In both the PEGASOS and the i/cDISCO protocol, in some cases unspecific fluorescence was observed. Particularly, prolonged incubation in DBE in i/cDISCO increased unspecific fluorescence in the stria vascularis, which got even stronger than the specific antibody staining over time (Supplementary Fig. S4 a).

The morphology of the organ of Corti was preserved in i/cDISCO and ECi protocols without obvious distortions, while PEGASOS often caused deformation of the organ of Corti structure, resulting in wavy hair cell rows (Supplementary Fig. S4a). Notably, the ECi protocol demonstrated the greatest reliability in terms of specific antibody labelling, staining continuity throughout the sample, and signal-to-background quality between experiments. Further, specific fluorescence intensity and low background were both stable for up to several weeks in ECi solution.

One advantage of water-based clearing protocols is that they better preserve tissue morphology by avoiding dehydration-induced shrinkage. Particularly in organs with complex mixtures of soft and hard tissues such as the cochlea, water-based strategies might thus be preferable to avoid shrinkage artifacts reported previously. To test water-based techniques, we chose Sca*l*eS and FRUIT [20, 21]. Sca*l*eS was selected because it had previously been adapted for cochlear clearing, whereas FRUIT was included as an additional hydrophilic, fructose-based protocol using comparatively simple and non-toxic reagents. Both water-based approaches resulted in even lower levels of transparency than PEGASOS, the worst performing organic solvent-based protocol. Sca*l*eS and FRUIT both failed to render bone, stria vascularis and blood vessels transparent (Fig. 1a, row 2). The FRUIT solutions were further challenging to handle due to their high viscosity and tended to become turbid within a few days, which limited imaging quality.

Immunostaining continuity, immunofluorescence signals and signal-to-background quality appeared variable in repeated experiments using both protocols. In more than half of the experimental trials, specific immunofluorescence was absent after Sca*l*eS clearing, and only rarely presented strong staining from apex to base (Fig. 1 b and Supplementary Fig. S4a).

Thus, compared to organic solvent-based protocols, the water-based clearing methods did not reduce light scattering to the same extent, resulting in more pronounced blurry regions in the images. Additionally, we observed more unspecific antibody binding and autofluorescence of the cochlear bony shelf in water-based protocols. Similar as in i/cDISCO and ECi, we did not observe morphological alterations in Sca*l*eS and FRUIT experiments (Fig. 1b and Table 1). Notably, in both Sca*l*eS and FRUIT, the agarose did not remain as a solid block, which made handling and mounting for imaging difficult.

In summary, i/cDISCO and ECi achieved the best transparency combined with good immunostaining continuity, signal strength and morphological preservation (Table 1), at the expense of long protocol durations.

### Optimization of Pre-processing Steps for Accelerated Tissue Clearing

**Fig. 2:**
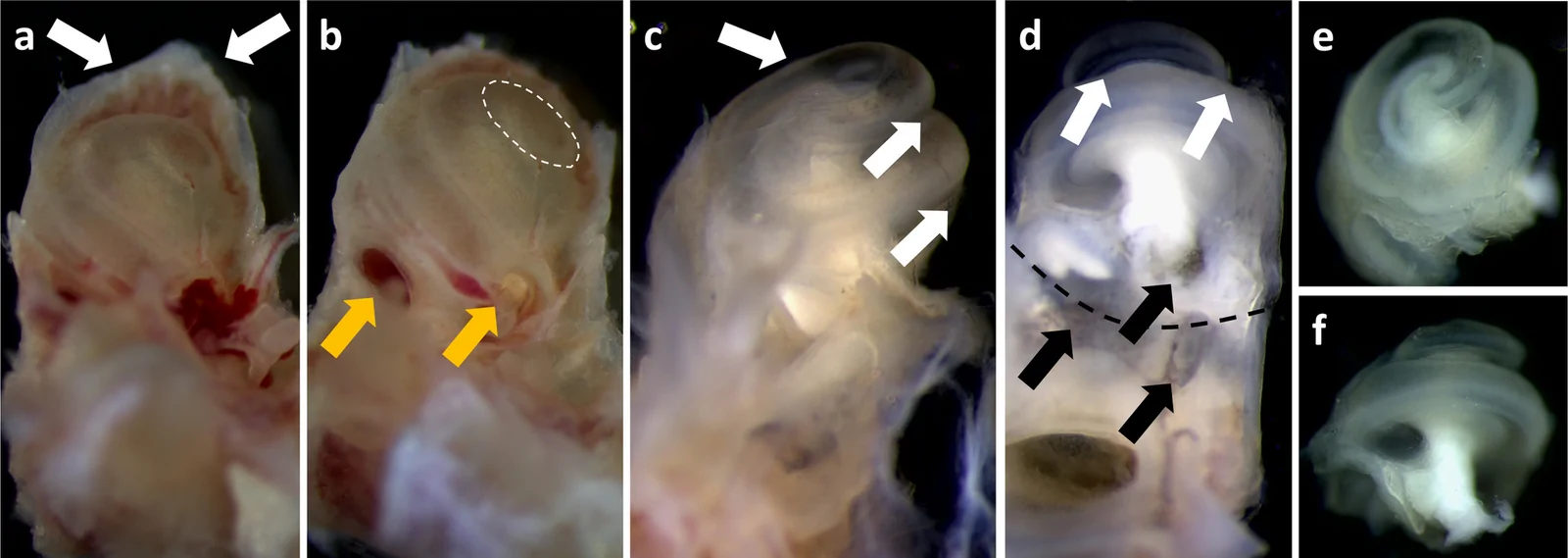
Cochlear trimming to allow faster clearing. **a** Cochlea after dissection from the skull. The white arrows indicate the highly pigmented bone connecting the cochlea with the bulla. **b** Cochlea after removal of the pigmented bony shelf. The white dotted line gives an impression of how big the hole at the cochlear apex should be for the fixation solution to enter by diffusion. The yellow arrows indicate where the oval and round window are gently punctured to ensure diffusion of solutions. **c**, **d** White arrows depict where extra removal of the bony shelf at the front (**c**) and back (**d**) of the cochlea is recommended to further shorten the decalcification time. After carefully separating the excessive bone and vestibular organ with sharp forceps (dotted line), remaining blood vessels (black arrows) should be detached. **e**, **f** Fully trimmed cochlea for one-day calcification and fast clearing

To accelerate the clearing process, we reduced the time required for decalcification, decolorization and refractive index matching. One key step is to remove the rather thick and pigmented part of the bony shelf at the back of the cochlea which connects to the bulla (Fig. 2a + b and Supplementary Fig. S2). Removal of this bone at the apical part of the cochlea allows a drastic reduction of the decalcification and decolorization steps and is crucially done before fixation and decalcification to avoid breaking of the cochlea. Subsequently, the fixation is performed as described, involving the diffusion of solutions through a hole in the apical cochlea and punctured oval and round window membranes.

We next optimized decalcification conditions by varying EDTA concentration, temperature and incubation time, using tissue handling, macroscopic bone softening and subsequent light-sheet imaging quality as practical endpoints. Increasing the EDTA concentration from 200 to 500 mM did not result in an obvious improvement in bone softening or subsequent clearing. In contrast, increasing the temperature during EDTA incubation strongly accelerated bone softening. At 4 °C, decalcification proceeded slowly and required several days to complete. We next compared 37 °C to room temperature (RT) and found that incubation at 37 °C for three days enabled sufficient decalcification for clearing and imaging. Prolonging the incubation in EDTA to 14 days did not further improve clearing results. Based on these observations, all subsequent experiments were performed using 200 mM EDTA at 37 °C.

To further shorten the decalcification time, we removed additional bone structures. This advanced trimming step requires more technical skills but can be omitted if maximal morphological preservation is prioritized over protocol speed. After a short EDTA pre-incubation at 37 °C (Supplementary Table S2), we removed approximately two thirds of the posterior bony shelf of the cochlea while aiming to preserve the underlying soft tissues (Fig. 2c + d). This optional pre-processing routine reduced the time required for sufficient decalcification from three days to 24 h. We further trimmed the semicircular canals and surrounding tissue including blood vessels (both for five- and seven-day protocols, Fig. 2d–f).

### Shortening Whole-Mount Antibody Staining to Accelerate the Protocol

**Fig. 3:**
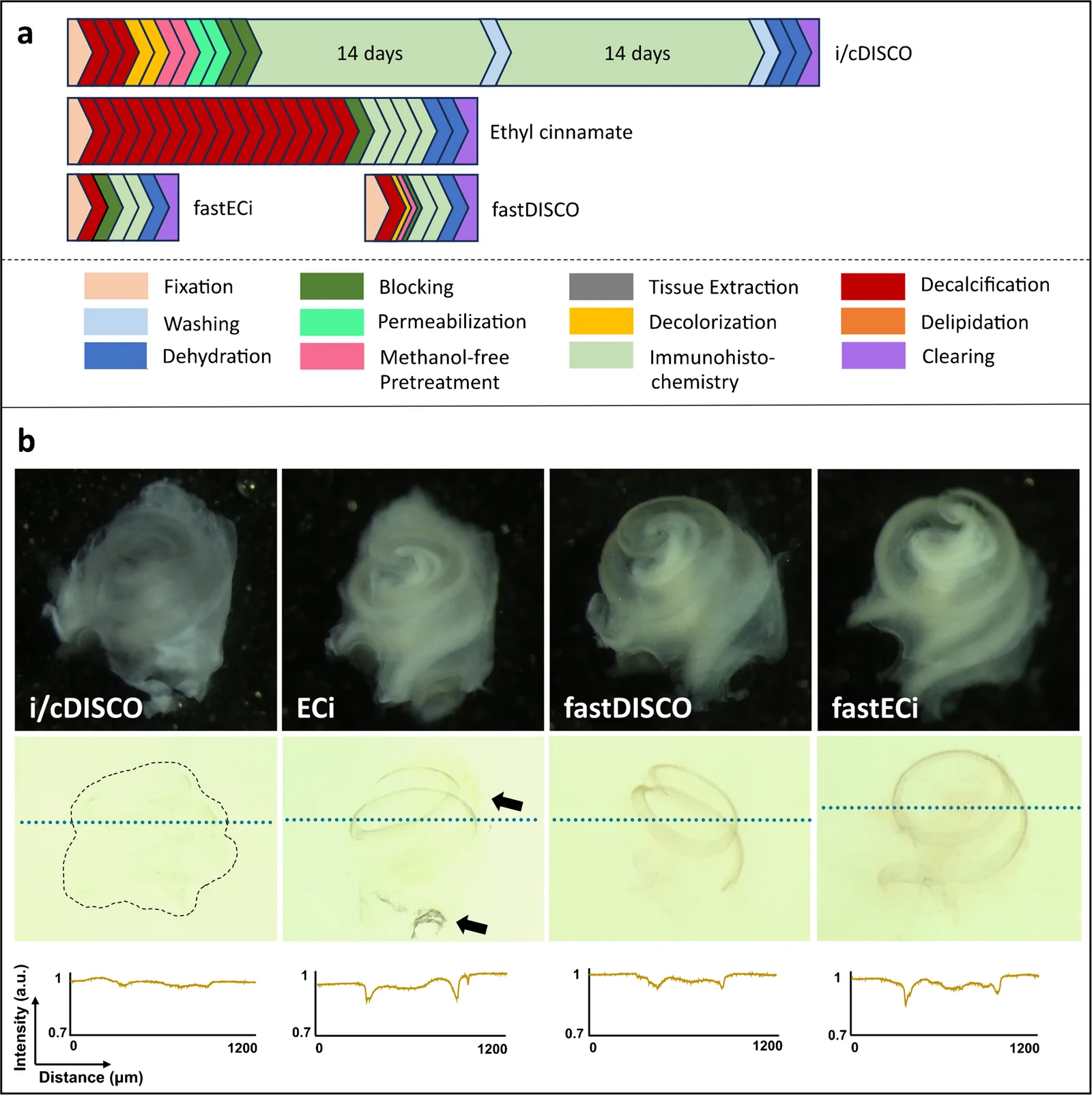
Schematic overview of original-length and fast cochlear clearing protocols and comparison of resulting transparency. **a**: Schematic depiction of the individual processing steps and step durations for the original-length protocols (i/cDISCO: appr. 44 days; ECi: appr. 26 days) and the optimized fast clearing protocols (fastDISCO and fastECi: 5 days). **b**, top row: cochleae from 2–3 month old mice prepared with the trimming strategies used for original-length and fast protocols. Middle row: the same cochleae after clearing with original and fast protocols, illustrating differences in optical transparency (see also Supplementary Fig. S3). The dashed black line outlines the i/cDISCO-cleared cochlea. Arrows in the ECi condition mark bone and blood vessels that remain visible after clearing. The blue dotted lines indicate the approximate position used for transparency assessment. Bottom row: line profile analysis of transparency (see also Supplementary Fig. S3)

To accelerate the two best-performing clearing protocols, i/cDISCO and ECi, we systematically reduced individual protocol steps (Fig. 3a) and evaluated the impact on transparency, antibody staining and morphology in 3D light-sheet reconstructions.

For i/cDISCO, we reduced the fixation time in 4% PFA from overnight to 3 h, which was sufficient to preserve the overall cochlear structure for downstream clearing and light-sheet imaging under the conditions tested. The decolorization step using Quadrol, which reduces autofluorescence and absorption from heme [2, 9], was shortened from 2 days to 2 h. Under our experimental conditions, this shorter incubation reduced residual absorption to a level compatible with light-sheet imaging of adult mouse cochleae. Although residual pigmentation of the stria vascularis was occasionally observed after fastDISCO clearing, it did not obviously prevent visualization of the organ of Corti in our datasets (Fig. 3b, middle row). Extended storage of cleared cochleae in DBE for >1 week further reduced the remaining pigmentation in the stria vascularis, rendering the cochlea completely transparent. However, this seemed to correlate with increasing autofluorescence of the stria, similarly as described for the original length i/cDISCO (see Supplementary Fig. S4 a and S5 a).

**Fig. 4:**
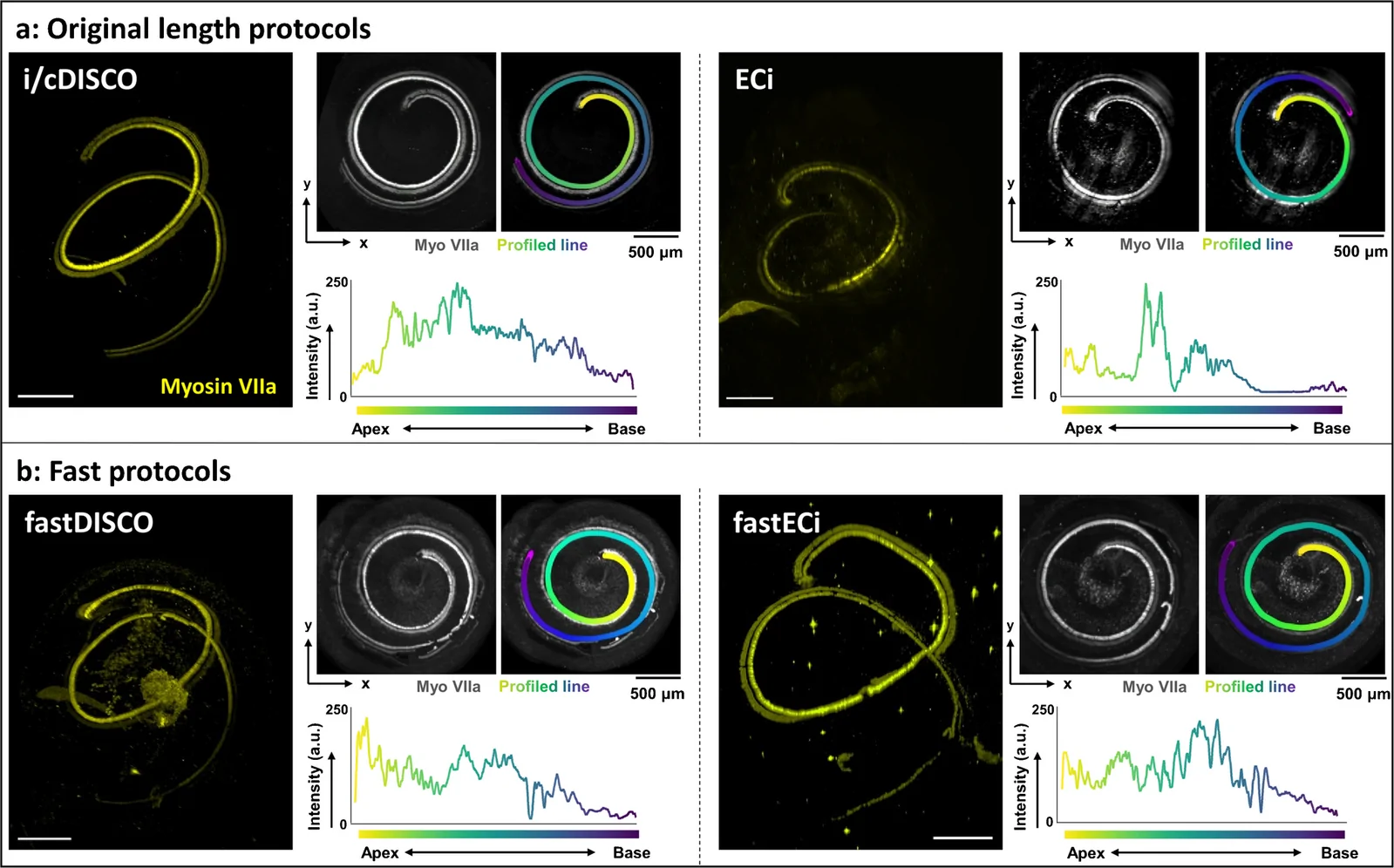
Myosin VIIa immunolabeling along the organ of Corti in original-length and fast clearing protocols. In representative cochleae from 2–3 month old (i/cDISCO, ECi, fastDISCO) or 7 month (fastECi) old mice, fluorescence intensity was extracted along a manually defined polyline tracing the hair cell row from apex to base and visualized using a color-coded profile (apex: yellow to base: purple). The same color coding is overlaid onto the top-view projections to enable spatial mapping of signal intensity to anatomical regions along the cochlear spiral. Cochleae were imaged with the apical side facing the detection objective, resulting in a gradual decrease in signal intensity toward the basal region due to light propagation through the tissue. **a** Original-length protocols. Left: i/cDISCO-cleared cochlea showing continuous myosin VIIa signal along the entire apical-to-basal extent of the organ of Corti. Right: ECi-cleared cochlea illustrating a representative example with local differences in the fluorescence signal along the cochlear spiral. **b** Fast clearing protocols. Left: fastDISCO-cleared cochlea, in this example with some unspecific fluorescence in the modiolus. Right: fastECi-cleared cochlea. In both cases, myosin VIIa signal is rather homogenously labelling hair cells along the full length of the organ of Corti. Scale bar: 300 µm

The original iDISCO protocol includes optional methanol-free pre-treatment taking approximately 130 h [15]. The cDISCO protocol recommends prolonged antibody incubation of 14 days for each primary and secondary antibodies [16]. This, together, accounts for the long duration of the combined i/cDISCO protocol. Omission of the methanol-free pre-treatment resulted in a markedly reduced immunofluorescence signal intensity. Consequently, we retained the original pre-treatment but shortened individual incubation steps, allowing completion within one day while still producing sufficient staining quality for volumetric light-sheet imaging under the conditions tested. In addition, antibody incubation times were reduced to overnight incubation for each step, which still resulted in continuous labelling of the organ of Corti in the optimized fast protocols (Fig. 4). To further assess whether shortened antibody incubation affects staining uniformity along the cochlear spiral, we assessed fluorescence intensity distributions along the length of the organ of Corti. For representative cochleae, three-dimensional light-sheet datasets were rotated into a standardized top-view orientation, and fluorescence intensity profiles were extracted along the hair cell row from apex to base using a manually traced polyline. Intensity values were normalized to cochlear length (0 = apex, 1 = base) to allow direct visualization of spatial signal distribution. These apex-to-base intensity profiles demonstrated staining continuity along the organ of Corti in both fastDISCO (Fig. 4b, left) and fastECi protocols (Fig. 4b, right).

After immunolabelling, methanol was replaced by ethanol for dehydration without negatively affecting clearing performance. With these adjustments and the advanced trimming of the cochlear bone, the total duration of the i/cDISCO protocol was reduced to five days (fastDISCO). The protocol with simplified trimming requiring three days of decalcification takes seven days in total (see Supplementary Fig. S2).

In the original ECi clearing protocol, decalcification represented the most time-consuming step. As shown above, 24-h incubation in 200 mM EDTA at 37 °C turned out to be sufficient for decalcification for the extensively trimmed cochlea. We further adjusted downstream steps by shortening blocking and antibody incubation as we did for fastDISCO. Dehydration was performed as in fastDISCO using a graded ethanol series with one hour incubation time for each step. Again, the thoroughly trimmed cochlea requires a total protocol duration of five days for complete clearing, the less trimmed cochlea can be cleared within seven days with fastECi (Fig. 3 and Suppl. Fig. S2, S6b).

### Validation of Morphological Preservation and Immunofluorescence of Cochlear Cells in Fast Protocols

**Fig. 5:**
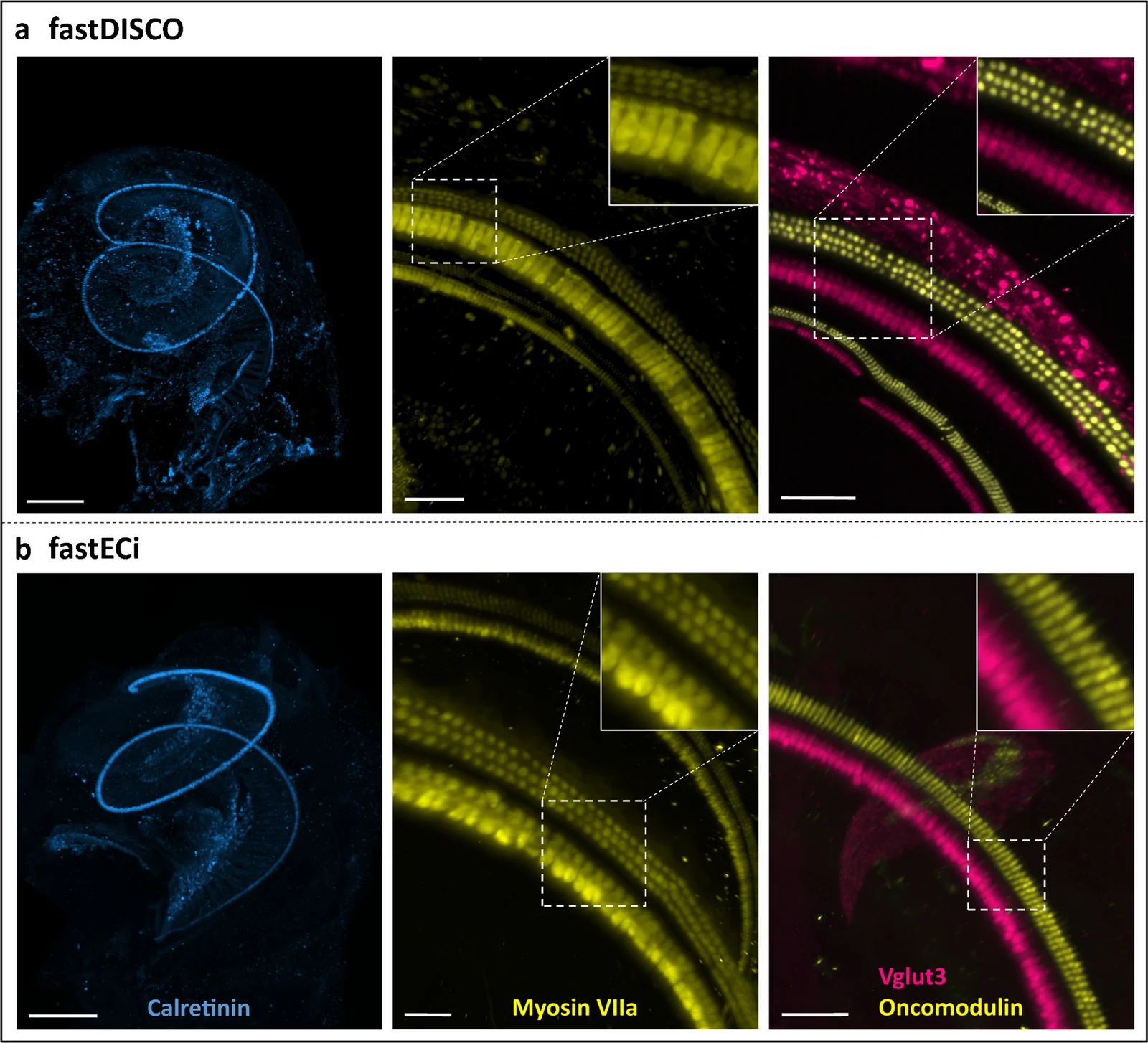
Immunolabeling of different hair cell markers in fast clearing protocols. Representative fastDISCO- (**a**) and fastECi-cleared (**b**) cochleae labeled with multiple hair cell markers. Inner hair cells are visualized using Vglut3, calretinin and parvalbumin, while outer hair cells are labeled using oncomodulin; myosin VIIa labels both inner and outer hair cells. Representative examples are shown from independent cochleae stained with different hair cell marker combinations: fastDISCO, *n* = 17; fastECi, *n* = 20; age of animals displaying calretinin immunolabelling: 1 month; other examples: 2–3 months. Scale bar in left panels: 300 µm. Scale in middle and right panels: 100 µm

To validate preservation of epitopes and morphology in the fast clearing protocols, we tested a panel of antibodies targeting key cochlear cell types. For hair cell labeling, vesicular glutamate transporter 3 (Vglut3), calretinin (Calb2) and parvalbumin were used as inner hair cell (IHC) markers, while oncomodulin reliably labeled outer hair cells (OHC). Myosin VIIa was used to robustly label both IHCs and OHCs (Figs. 4 and 5). In total, we combined three different markers per sample (Fig. 5 and Suppl. Fig. S5). In addition, SYTOX™ Green was used as a nuclear counterstain to support visualization of nuclei and tissue organization during image analysis. (for further details on antibody stainings see Supplementary Table S1).

Both fastDISCO and fastECi resulted in homogeneous staining along the entire apical-to-basal axis of the organ of Corti for all markers, allowing reliable detection of individual hair cells, but also their loss (Figs. 4a, b and 5a, b; Suppl. Fig. S5 and Fig. 7b).

**Fig. 6:**
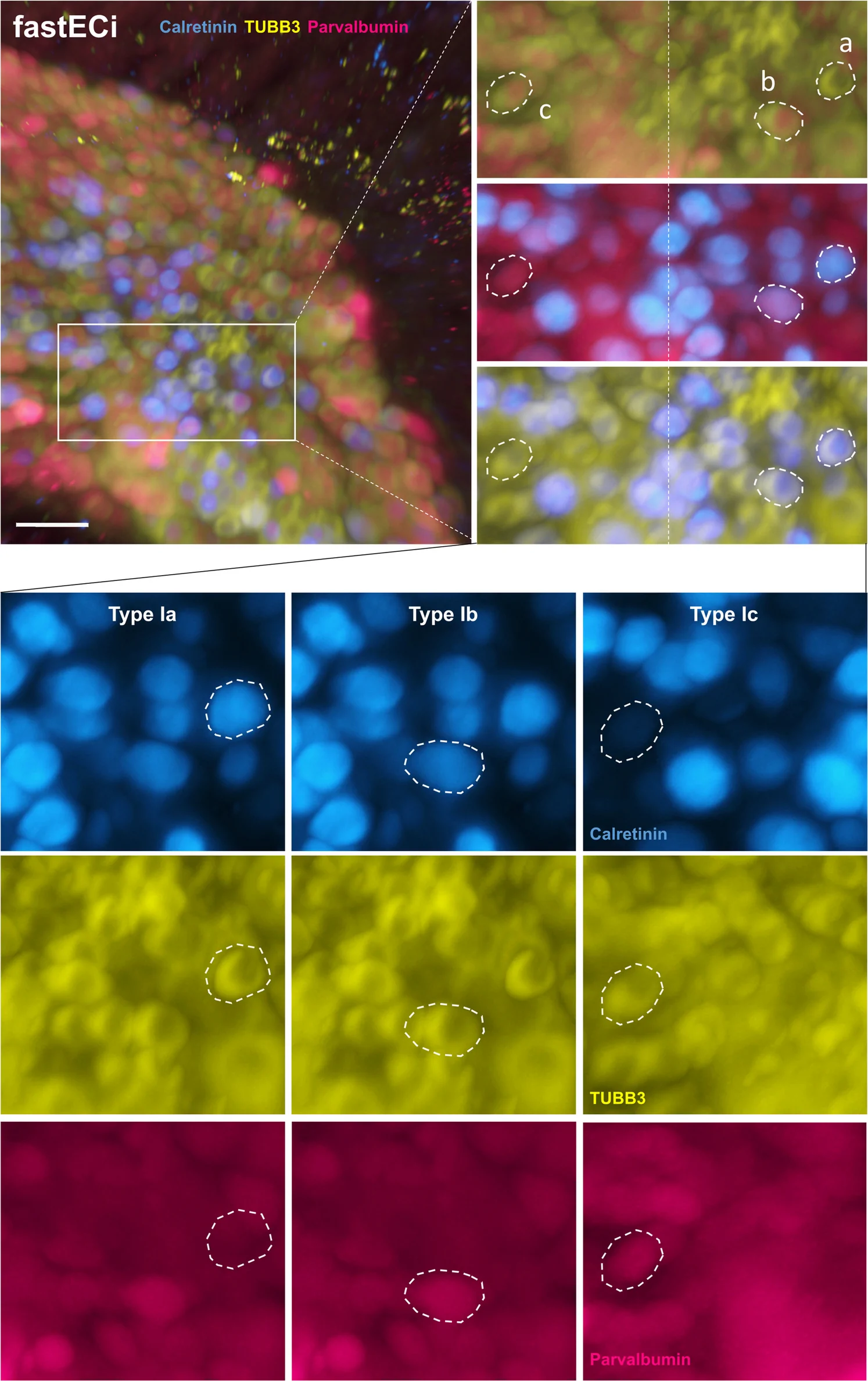
Spiral ganglion neuron (SGN) immunostaining in fastECi. Representative fastECi-cleared cochleae immunolabeled for SGN subtypes. Anti-tubulin β-III (TUBB3) labels all SGNs, while calretinin and parvalbumin selectively label type I SGN subtypes. Calretinin shows strongest labeling of type Ia SGNs, parvalbumin labels type Ic SGNs, and type Ib SGNs are labelled with both markers. Upper panels: merged images. Lower panels: higher-magnification views of the indicated regions, illustrating distinguishable SGN subtypes; dotted lines outline the respective SGN subtypes. Representative example of *n* = 20 independent fastECi-cleared cochleae. Scale bar: 50 µm

Spiral ganglion neurons (SGNs) and associated nerve fibers were examined using antibodies against tubulin beta-III (TUBB3), calretinin and parvalbumin. TUBB3 labeled neurites of all SGN types, whereas calretinin and parvalbumin selectively labeled type I SGNs, which can be further divided into subtypes Ia, Ib and Ic [22]. Using calretinin and parvalbumin immunostaining, differentiation between SGN subtypes Ia, Ib and Ic was possible in fast-cleared samples (Fig. 6, Supplementary Movie 1). Notably, even in a sample with partial rupture at the basal SGN region, SGN-associated labelling remained detectable on both sides of the rupture (black rift between SGNs, Supplementary Fig. S5 b, Supplementary Movie 1). Yet, unspecific staining of SGNs was detected in some cochleae after fastDISCO clearing (Fig. 4b, left).

In both, fast and original DISCO-based protocols, we occasionally observed autofluorescence in the stria vascularis, predominantly in channels corresponding to the excitation wavelengths of 647 nm and 790 nm. Particularly with the latter, we noticed an unspecific stria vascularis (auto-)fluorescence in almost all fastDISCO or i/cDISCO cleared cochleae as soon as samples were stored in DBE solution for more than 1–2 weeks (Supplementary Fig. S4a and S5a). However, when imaging was performed within one week after clearing, no such autofluorescence was detected.

To assess a potential improvement of stria vascularis transparency by attempting to wash out remaining blood from the vessels prior to clearing, we tested cochleae from transcardially perfused animals and compared these to non-perfused cochleae after clearing with the fastECi protocol (Supplementary Fig. S7). Since we observed no obvious difference in terms of tissue transparency, we assume that transcardial perfusion does not lead to substantial removal of blood from tiny blood vessels in the stria vascularis and is thus likely not superior to our standard procedure without transcardial perfusion.

Overall, morphological distortions, like wavy appearance of the basilar membrane, were not observed in fast protocols, similarly to original-length protocols, indicating that protocol shortening does not compromise tissue integrity.

### Fast Clearing Protocols Support Hair Cell Row Segmentation and Hair Cell Counting

**Fig. 7:**
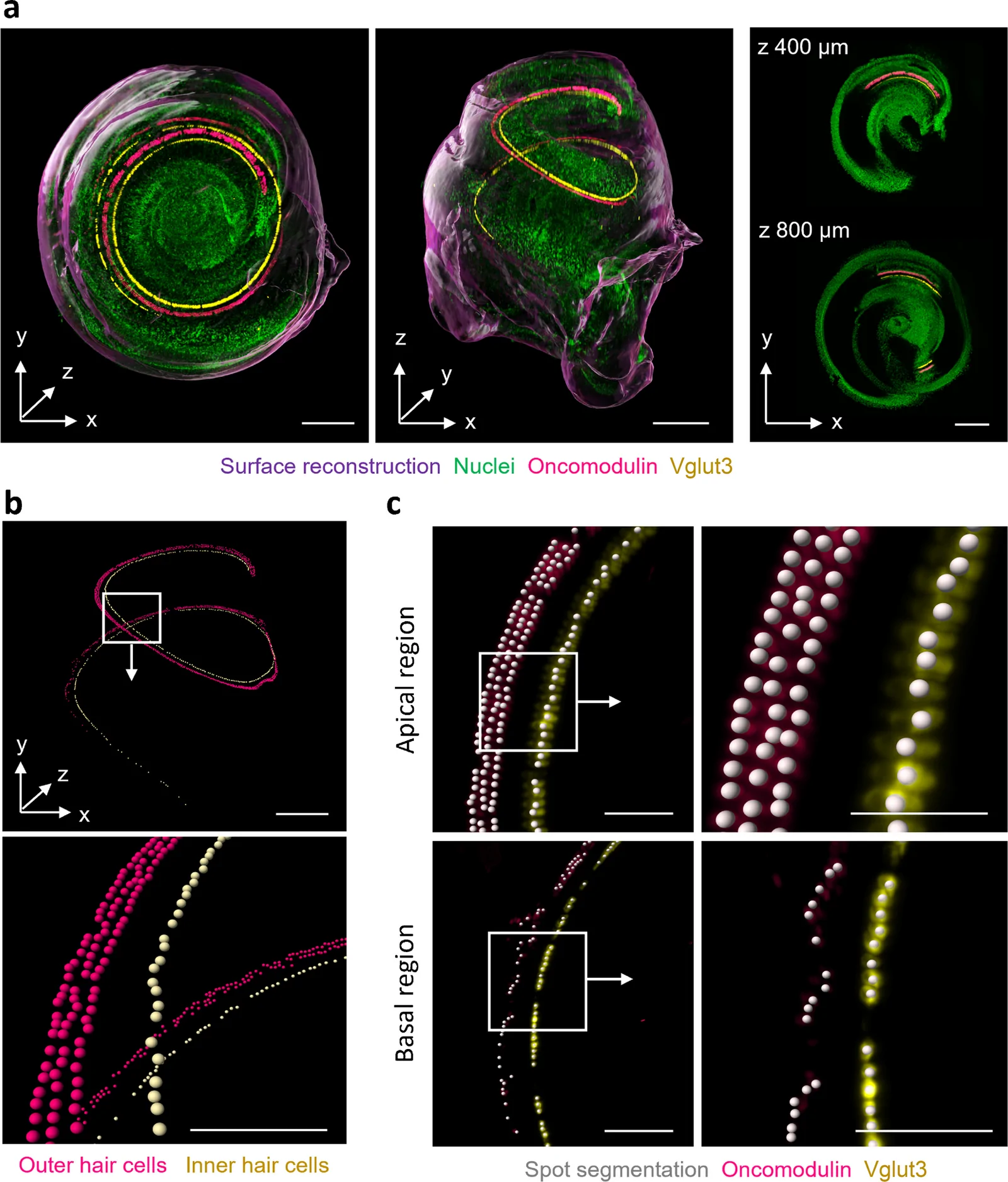
Segmentation of cochlear hair cell rows and cell counting is supported by fast clearing protocols. **a** Three-dimensional light-sheet microscopy reconstructions of a representative fastECi cleared cochlea immunolabeled for nuclei (green), Oncomodulin (magenta) and Vglut3 (yellow). A surface reconstruction of the cochlea is shown in purple. Selected *z*-planes (right panel) illustrate preservation of tissue architecture and signal quality throughout the sample depth. **b** Segmentation of inner and outer hair cell rows based on hair cell markers. The IHC row extends beyond the OHC marker signal, consistent with focal OHC loss in aged mice. **c** Higher-magnification view of hair cell detection using spot segmentation. Apical regions (top) show regular inner and outer hair cell rows while basal regions (bottom) show pronounced hair cell loss in a 9-month old C57BL/6 mouse. Representative segmentation example of *n* = 5 segmented fastECi-cleared cochleae

To assess whether the optimized fast clearing protocols support automated, object-based quantitative analysis, we performed three-dimensional segmentation of cochlear hair cells in a representative fastECi-cleared cochlea. Segmentation was based on combined nuclear counterstaining and immunolabeling for Vglut3 and oncomudulin, enabling identification of individual inner and outer hair cells within the intact organ of Corti (Fig. 7a–c).

The resulting segmentation allows visualization of hair cell distributions throughout the cochlear spiral and enabled extraction of quantitative readouts, such as object counts along the apical-to-basal axis. While the present analysis is based on a single representative dataset and is not intended for statistical comparison, it demonstrates that the signal quality and structural preservation achieved with the fast clearing workflows are sufficient to support automated, annotation-free segmentation and quantitative analysis of cochlear cell populations.

### Comparison and Overall Outcome of Original and Fast Protocols

In total, we systematically compared five established clearing protocols and optimized the two best-performing protocols. To enable an integrated comparison of all seven protocols, we assessed multiple attributes required for effective cochlear clearing and complemented these with criteria reflecting practical laboratory feasibility (e.g., toxicity of reagents or protocol duration). Seven categories were defined and scored on a scale from zero (lowest) to four (highest). Notably, we weighed all categories equally (see Table 2).

**Table 2:** Qualitative evaluation of seven clearing protocols

	i/cDISCO	fastDISCO	ECi	fastECi	PEGASOS	Sca*l*eS	FRUIT
Transparency	4	4	3	3.5	2	1	1^2^
Morphology	4	4	4	4	2.5	4	4
Immunostaining continuity	4	3.5	4	4	4	3	2
Signal-to-background quality	3^1^	2	3.5	4	2.5	2	2
Protocol length	1	4	2	4	2.5	3	3
Feasibility	2	2.5	4	4	3	3	1
Reliability	2	2	4	4	2.5	1.5	0.5
Total (max: 28)	20	24	24.5	27.5	18	17.5	13.5

^1^When imaged within 1–2 weeks after clearing; longer incubation in DBE leads to increased autofluorescence in the stria vascularis

^2^FRUIT solution turns turbid within a few days, further reducing the transparency

Samples were cleared according to the published protocols or the optimized five-day fast protocols. Transparency was assessed based on residual visibility of bone, blood vessels and stria vascularis pigmentation in brightfield images. Immunostaining continuity was assessed based on the presence of specific immunofluorescence labelling along the organ of Corti from apex to base, considering major unlabelled regions and signal dropouts. Signal-to-background quality was assessed qualitatively based on the brightness of specific antibody labelling compared with nonspecific fluorescence or autofluorescence. Morphological preservation was evaluated based on visible distortion of the organ of Corti. Protocol length was scored based on the total duration of the workflow, and feasibility was evaluated based on practical aspects such as reagent toxicity, handling complexity, number of processing steps and solution stability. Lastly, reliability determined the comparability of outcome between different clearing rounds. Clearing protocols were evaluated across seven categories, each scored up to four credits, with all categories weighted equally. Scores summarize the overall evaluation of those independent cochleae where some immunolabelling could be detected; reliability considers all independent experiments: i/cDISCO, *n* = 10; fastDISCO, *n* = 17; ECi, *n* = 10; fastECi, *n* = 20; PEGASOS, *n* = 10; Sca*l*eS, *n* = 8; FRUIT, *n* = 4

FRUIT, Sca*l*eS, and PEGASOS achieved lower overall scores, although for different reasons. These included variability between clearing trials, morphological alterations, inconsistent immunofluorescence labeling or limited solution stability. In contrast, the original i/cDISCO and ECi protocols performed well across most criteria, with protocol duration representing their primary constraint. This limitation was effectively addressed by the optimization resulting in the respective fast protocols. Both fastDISCO and fastECi retained the key advantages of their original counterparts while substantially reducing protocol duration. Among all evaluated approaches, fastECi showed superior performance in terms of staining continuity, signal-to-background quality and reliability, resulting in the highest overall score and identifying fastECi as the most suitable protocol for routine cochlea tissue clearing.

## Discussion

Tissue clearing holds great promises for the analysis of complex 3D structures, however, different tissue types require specific adaptations of clearing methods. To date, only a limited number of studies have used tissue clearing for whole mount analysis of the complex helical organ of Corti within the bony cochlea. In this study, we systematically compared established clearing techniques and aimed to find the most suitable and feasible method for cochlear clearing in routine laboratory use. While comparing different clearing techniques, we noticed the differences in length for the same steps between the protocols, indicating that the respective shortest duration might be sufficient also in other approaches. We thus chose to further optimize the two best-performing protocols to accelerate cochlear clearing.

Most published cochlea clearing protocols rely on whole-animal perfusion for tissue fixation. However, in our hands, transcardial perfusion of the animals did not reduce transparency of the stria vascularis after cochlear clearing with fastECi (Suppl. Fig. S7). Since removal of large blood vessels and thick bony structures, for which this procedure does make a difference, can easily be implemented, transcardial perfusion is not further improving the clearing results of a trimmed cochlea.

Decalcification represented a major time-consuming factor across protocols. The thickest bone, which requires the most extensive decalcification and proved difficult to be fully decolorized, is the bony shelf at the posterior side of the cochlea (Fig. 2a). This bone can rather easily be trimmed (Fig. 2b and Suppl. Fig. S2) which is recommended to be done before decalcification. Using this pre-processed cochlea, we systematically evaluated EDTA concentration, incubation temperature and time, and found three days of incubation in 200 mM EDTA to be sufficient for complete decalcification and optimal clearing outcome when incubated at 37 °C. By targeted removal of about 2/3 of the cochlear bone surrounding the stria vascularis, the decalcification time could be further reduced to 24 h without compromising tissue clearing. However, while this advanced bone trimming might need practice, it is not absolutely necessary and can be replaced by three-day EDTA incubation of the cochlea and is thus feasible for less experienced users (see Suppl. Fig. S2, S6).

Mechanical agitation during clearing is frequently used to enhance diffusion of solutions. However, in our hands, shaking or rotation of cochleae resulted in tissue rupture, particularly affecting the organ of Corti. To preserve tissue integrity, we therefore omitted mechanical movement. While this choice may slightly prolong diffusion-dependent steps, it improved reproducibility and structural preservation. Future protocol adaptations may explore gentler agitation strategies to further reduce incubation times.

Across all tested protocols, major differences were observed in the feasibility of the protocol (toxicity, viscosity, and number of different solutions required), the resulting transparency, the reliability of immunofluorescence, and morphological integrity (Table 2). ECi-based clearing proved particularly robust, relying on readily available reagents and simple handling. In contrast, i/cDISCO required many reagents and buffer exchanges, while FRUIT was the most difficult to handle due to high viscosity and fast solution turnover. Although organic solvent-based clearing protocols were generally efficient in rendering the cochlea transparent and yielding strong and homogeneous immunofluorescence, some protocol-specific artifacts were observed: First, PEGASOS frequently induced a wavy deformation of the organ of Corti, likely due to tissue shrinkage (Suppl. Fig. S4). Second, i/cDISCO and fastDISCO occasionally resulted in unspecific stria vascularis staining at red/far-red excitation (647 nm and 790 nm) which appeared reliably after extended storage in DBE, limiting long-term sample stability. In addition, nonspecific SGN-associated fluorescence was occasionally observed in fastDISCO/i/cDISCO-cleared cochleae, reducing the reliability of specific SGN immunolabelling compared to fastECi. While such effects may be mitigated through further optimization of imaging parameters, they highlight protocol-dependent constraints that are particularly relevant for multicolor imaging applications.

During early imaging experiments, we noted that image quality was not solely determined by tissue transparency. In particular, differences between axial and lateral resolution in light-sheet microscopy led to variable sharpness depending on sample orientation. To overcome this, we embedded our samples in low melt agarose and ensured a consistent positioning, with the modiolus perpendicular to the optical pathway during imaging, which substantially improved image quality. Nevertheless, axial resolution remains a limiting factor for isotropic imaging of the cochlea. Emerging optical approaches, such as axially swept light-sheet microscopy, offer promising solutions but are not yet widely accessible [23].

Among all tested protocols, ECi, cDISCO and Sca*l*eS have previously been adapted for cochlea clearing [16, 17, 20]. Despite published reports of successful Sca*l*eS application, we were unable to reproduce comparable clearing quality and immunolabeling consistency [20]. Similarly, reconstruction of the cDISCO workflow was difficult and required combining descriptions from multiple sources [15, 16], resulting in the “i/cDISCO” protocol used in this study. Protocol-specific artifacts observed here, like autofluorescence of the stria after prolonged DBE incubation, were not reported previously. In contrast, ECi-based clearing consistently yielded robust results in terms of tissue transparency, immunofluorescence intensity and practicality. These strengths were retained or further enhanced in the optimized fastECi protocol.

Overall, fastECi combined short processing time with reliable clearing, homogeneous immunolabeling and excellent tissue preservation. Its robustness and ease of implementation make it particularly suitable for routine cochlear analysis and quantitative applications, including automated hair cell counting and large-scale comparative studies.

## Conclusion

We demonstrate that high-quality immunolabeling and complete cochlear clearing, which is compatible with 3D light-sheet microscopy and analysis, can be achieved within five days. Empirical shortening of fixation, decalcification and antibody incubation steps did not compromise tissue integrity or staining quality. The fast protocols support robust labeling of multiple cochlear cell types and will enable quantitative analyses across the entire organ of Corti, including hair cell row segmentation and cell counting. Among all evaluated approaches, fastECi combines reliability, practicality and reproducibility, making it well suited for routine cochlear clearing and downstream quantitative applications.

## Supplementary Information

Below is the link to the electronic supplementary material.

Supplementary file1 (MP4 99464 KB)

Supplementary file2 (PDF 861 KB)

## Data Availability

Data will be made available on reasonable request.
