# Supplementary material for: Comparing and Optimizing Tissue Clearing Protocols for Cochlear Immunostaining and 3D Reconstruction by Light-Sheet Microscopy
Source: J Assoc Res Otolaryngol. 2026 Aug 24;27(5):715–33. doi: 10.1007/s10162-026-01068-6 (PMC13687511; doi:10.1007/s10162-026-01068-6)
Supplement: Supplementary file 1 [file 10162_2026_1068_MOESM2_ESM.pdf]

Supplemental Information for:

Comparing and optimizing tissue clearing protocols for cochlear immunostaining and 3D reconstruction by light-sheet microscopy

JARO

Franziska M. K. Becker[1], Gina Dunkel[2,3], Martina Giampetraglia[2,3], Bettina Weigelin[2,3], Ellen Reisinger[1]

[1]Gene therapy for hearing impairment group, Dept for Otolaryngology – Head & Neck Surgery, University Hospital and Faculty of Medicine, University of Tübingen, Tübingen, Germany

[2]Werner Siemens Imaging Center, University Hospital and Faculty of Medicine, University of Tübingen, Tübingen, Germany

[3]Cluster of Excellence iFIT (EXC 2180) “Image-Guided and Functionally Instructed Tumor Therapies”, University of Tübingen, Tübingen, Germany

Correspondence:

Bettina.weigelin@med.uni-tuebingen.de

Ellen.reisinger@uni-tuebingen.de

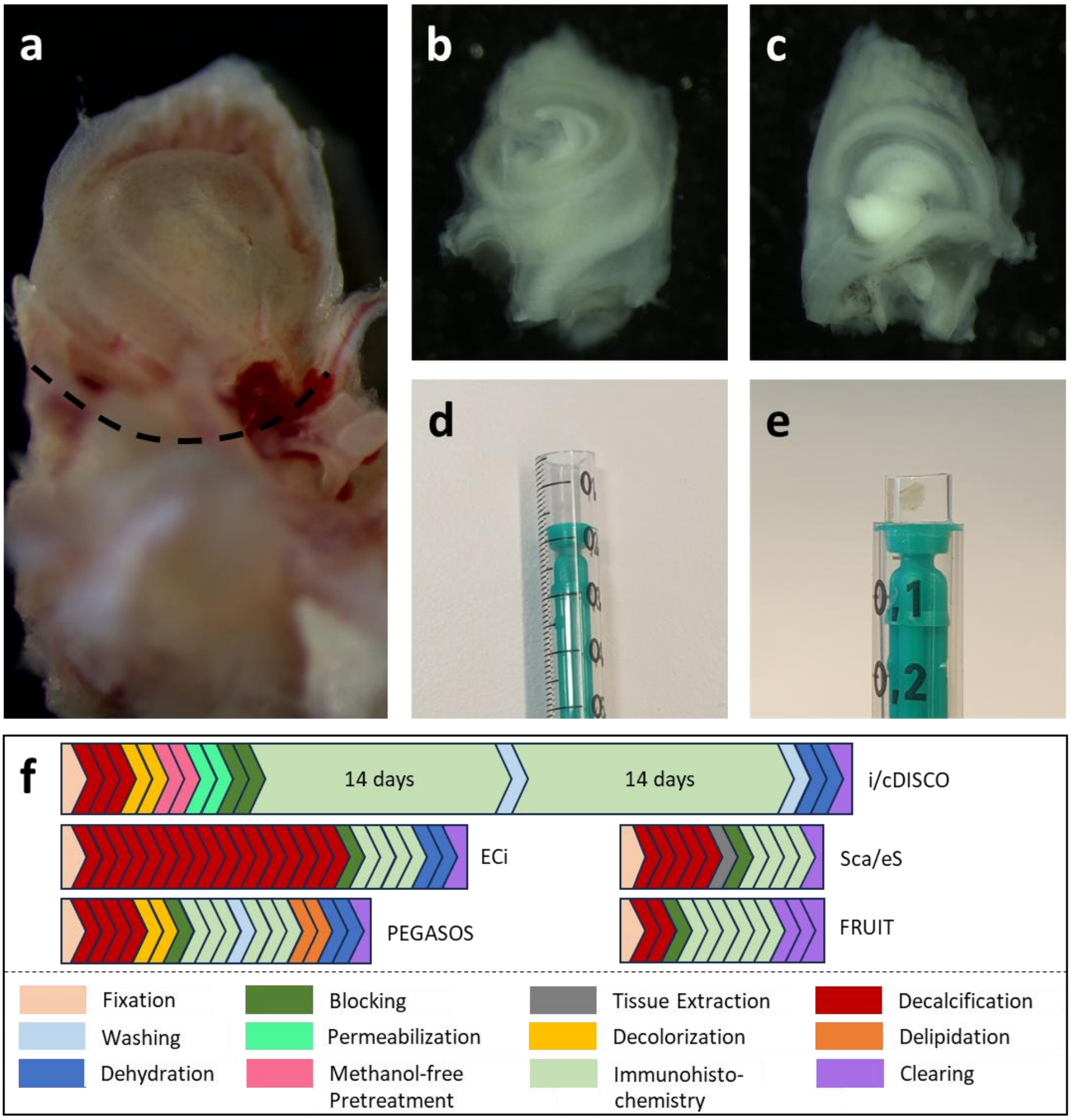

Fig. S1: Dissection of cochleae for original-length protocols, modification of a 1- ml syringe for agarose embedding, and schematic illustration of different steps in original protocols. a: Mouse cochlea as dissected from the temporal bone; The black dashed line indicates the removal of excessive bone, vestibular organ and semicircular canals. **b+c:** Trimmed cochlea before embedding in agarose. d**:** Cut-off of the nozzle of 1 ml-syringe for agarose embedding. **e:** Agarose block with embedded cochlea. **f:** Depiction of length and different steps of original clearing protocols. Duration of individual protocols: i/cDISCO: appr. 44 days, ECi: appr. 26 days, PEGASOS: appr. 20 days, Sca*l*eS and FRUIT: appr. 13 days.

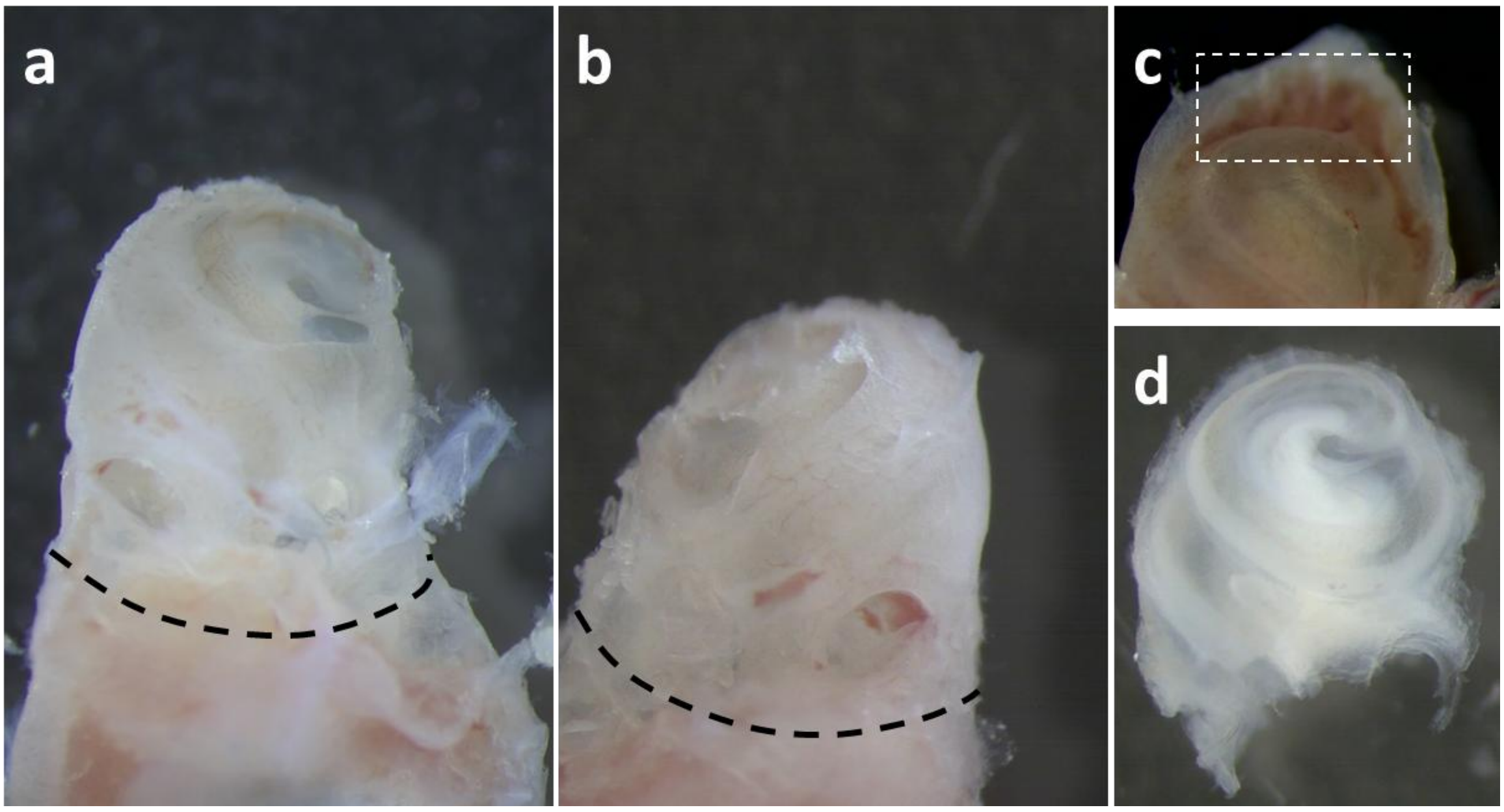

Figure S2: Minimum of dissection needed for decalcification in three days. a+b: The bony shelf connecting the cochlea with the bulla needs was removed completely. Dotted line indicates where the vestibular organ should be removed before decalcification. **c:** Pigmentation of the inside of the bony shelf and reason for removal. d**:** Cochlea after decalcification.

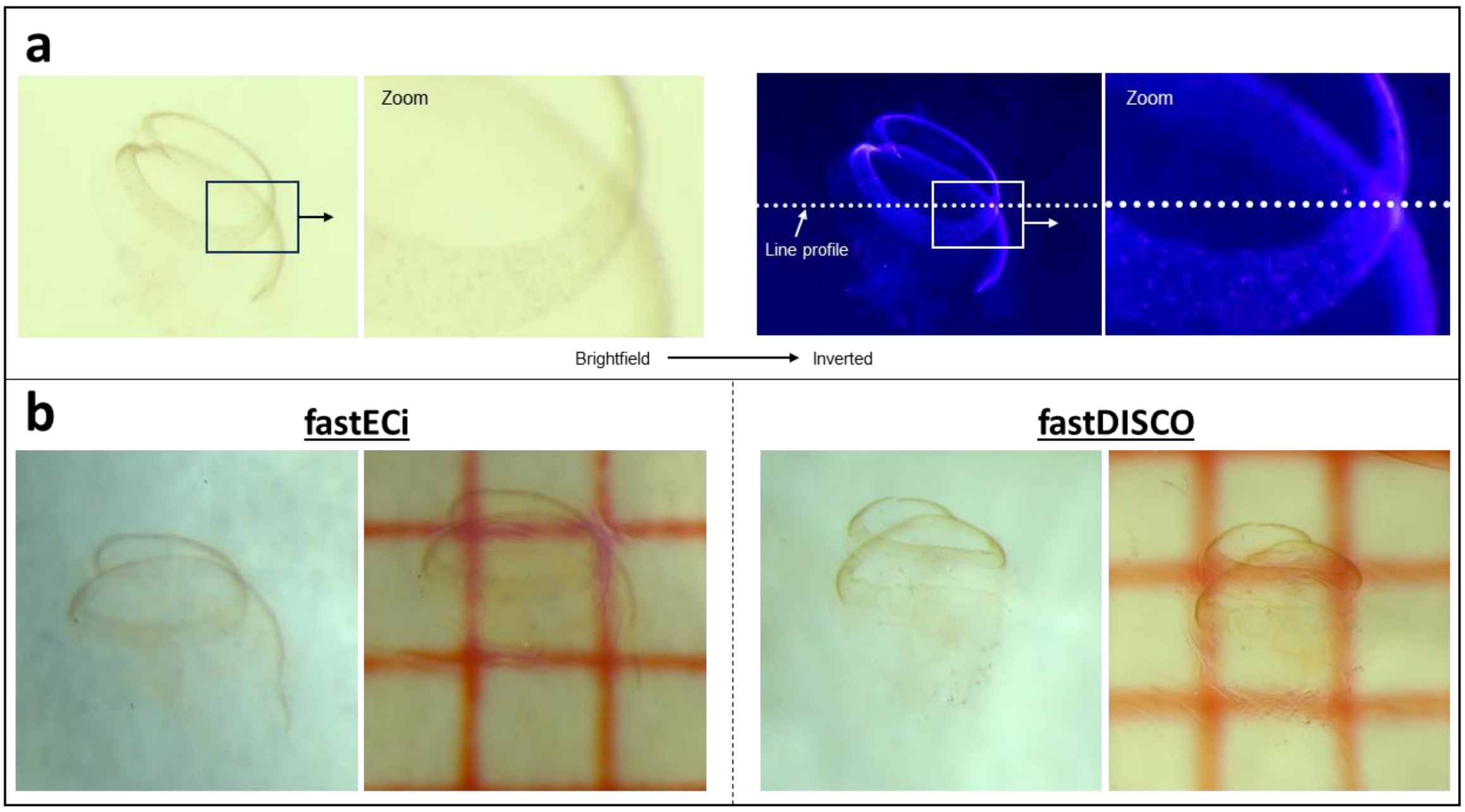

Figure S3: Visualization of the transparency line-profile analysis workflow. a: Representative brightfield image of a fast-cleared cochlea used for transparency assessment, with a higher-magnification view shown on the right. To facilitate visualization of residual light absorption and scattering, the brightfield image was converted to an inverted grayscale representation prior to line profile extraction. The dotted line indicates the position of the line profile used to extract grayscale intensity values across the tissue, as shown in Figure 1. **b:** Illustration of transparency on graph paper as background.

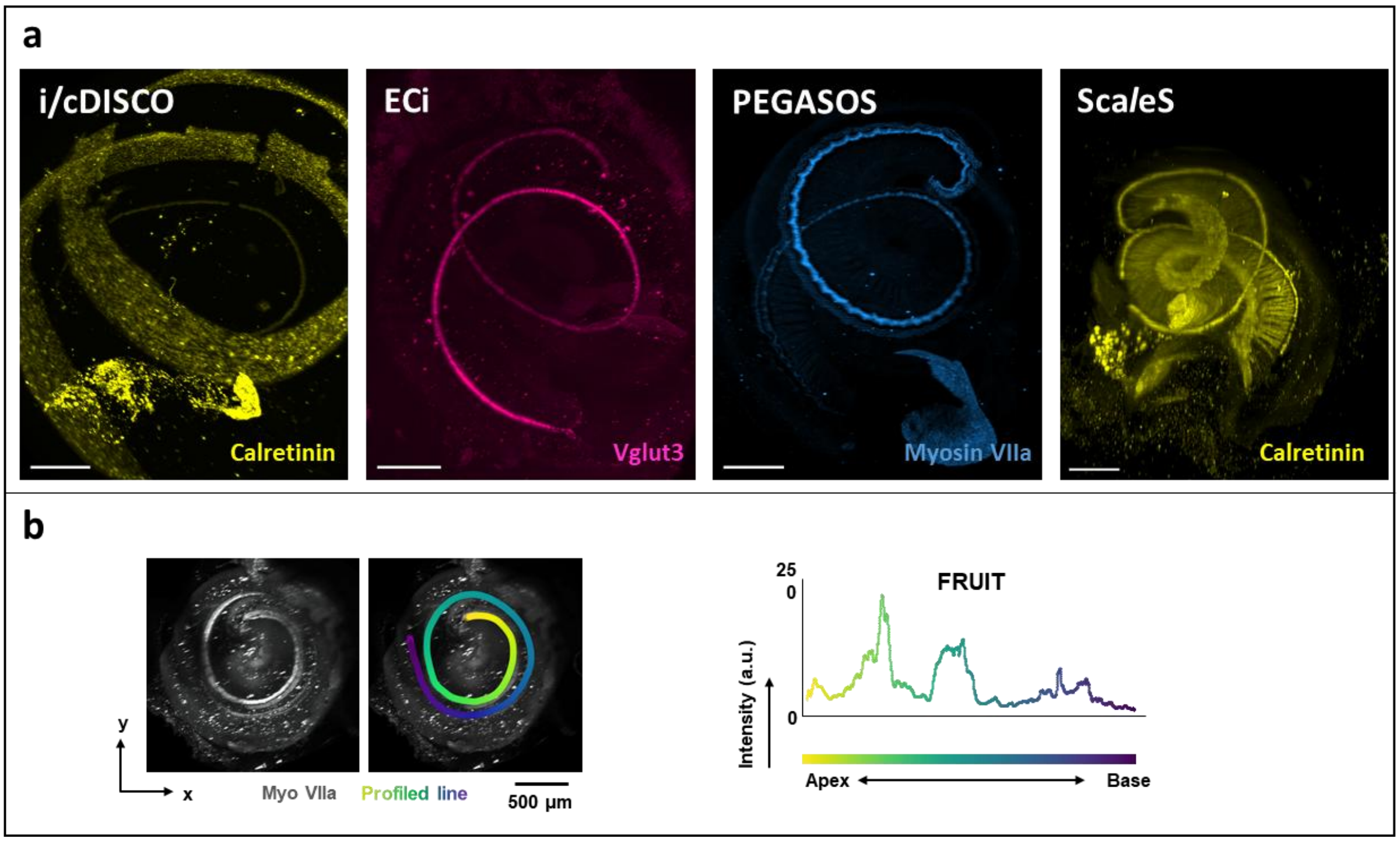

Figure S4: Examples of variable outcomes observed with original-length clearing protocols. a: Three-dimensional light-sheet microscopy reconstructions from independent experimental repetitions using original-length clearing protocols and the indicated antibodies. Images illustrate variability in immunofluorescence signal intensity and distribution between trials. Scale bar: 300 µm. Color code: blue, secondary antibody detected at 594 nm, yellow, secondary antibody detected at 647 nm, magenta, secondary antibody detected at 790 nm. **b:** Representative example illustrating spatial variability of myosin VIIa immunofluorescence along the organ of Corti in samples cleared using the FRUIT protocol. **Left:** top-view projection with the polyline used for fluorescence intensity profiling overlaid. **Right:** corresponding apex-to-base fluorescence intensity profile, visualizing local variations in signal intensity along the cochlear spiral. Cochleae in the representative images were isolated from animals of different ages; i/cDISCO = 6 months, ECi = 2.4 months, PEGASOS = postnatal day 36, Sca*l*eS = 4 months.

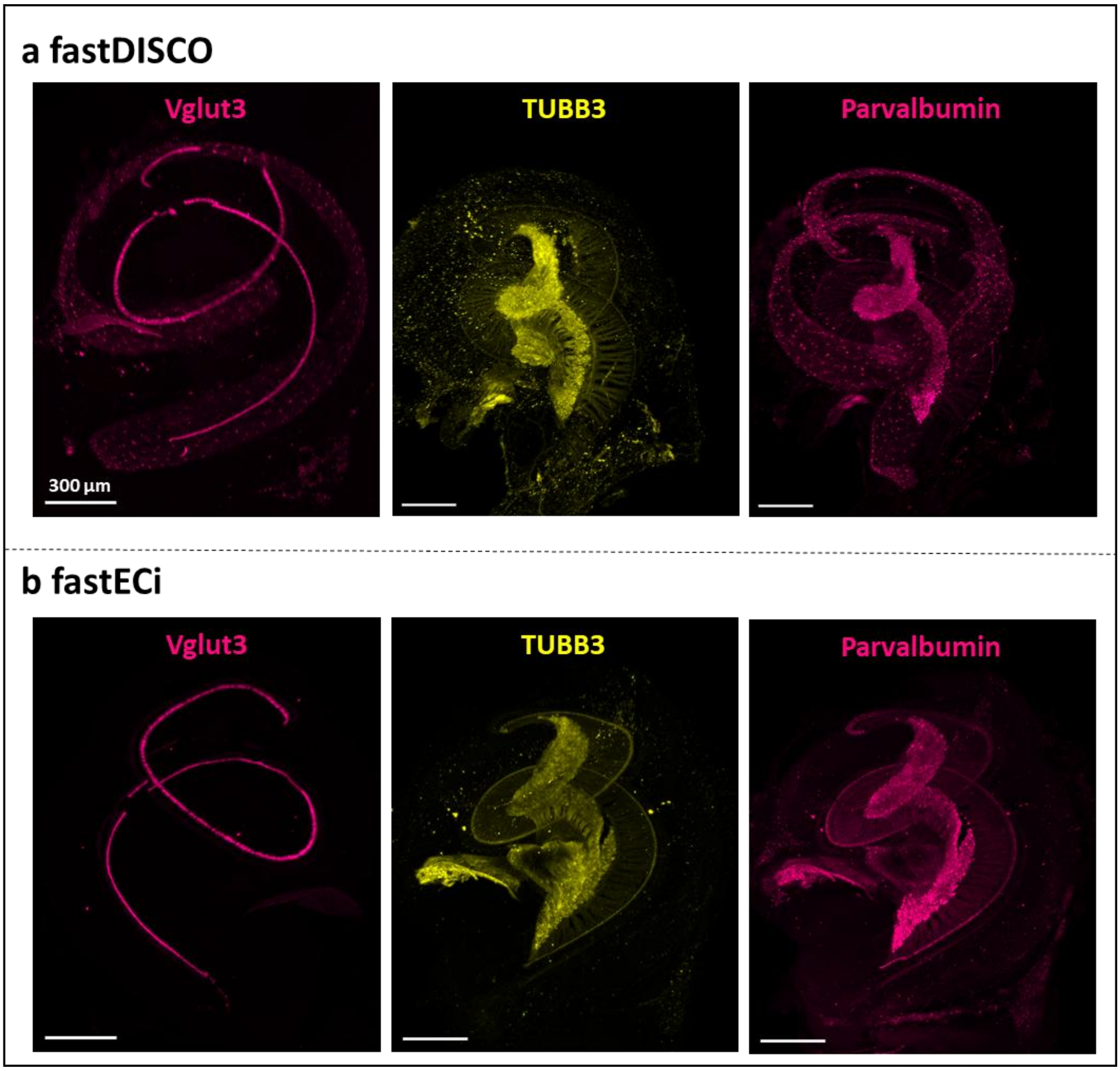

Fig. S5: Exemplary application of different antibodies during fast protocols. Representative fastDISCO- (**a**) and fastECi-cleared (**b**) cochleae immunolabeled with Vglut3, TUBB3 and parvalbumin, illustrating compatibility of the fast protocols with multiple antibody stainings. Scale bar: 300 µm. Animals for the representative cochlear images were of different ages; fastDISCO = postnatal day 34, fastECi (Vglut3) = 3 months, fastECi (TUBB3, Parvalbumin) = postnatal day 34.

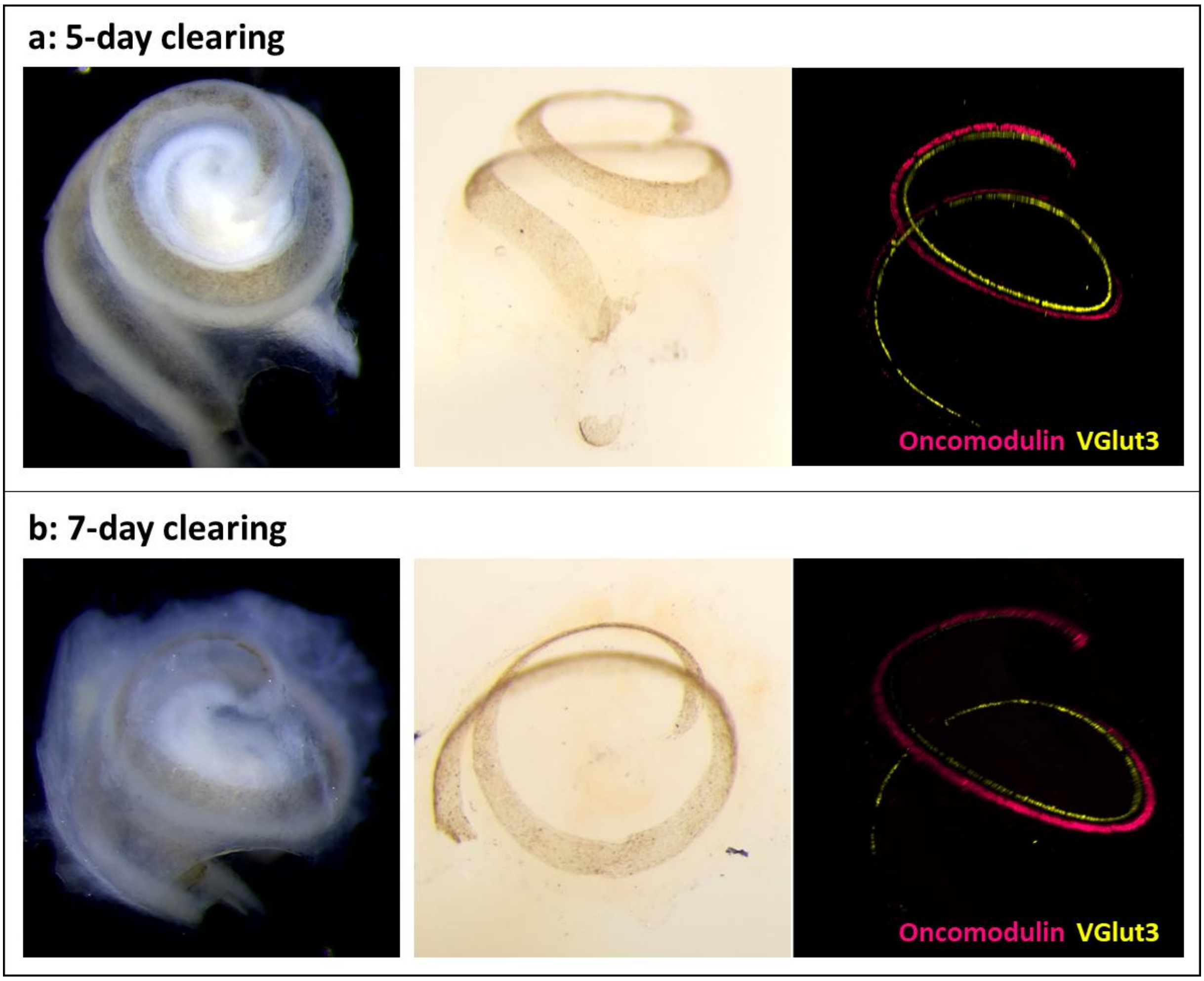

Fig. S6: Representative examples of five-day (a) and seven-day clearing (b) with fastECi. Oncomodulin (magenta) labels outer hair cells and Vglut3 (yellow) inner hair cells. Age of animals: 9 months – note the decline of outer hair cells towards the base, which is typical for C57BL/6 mice at that age.

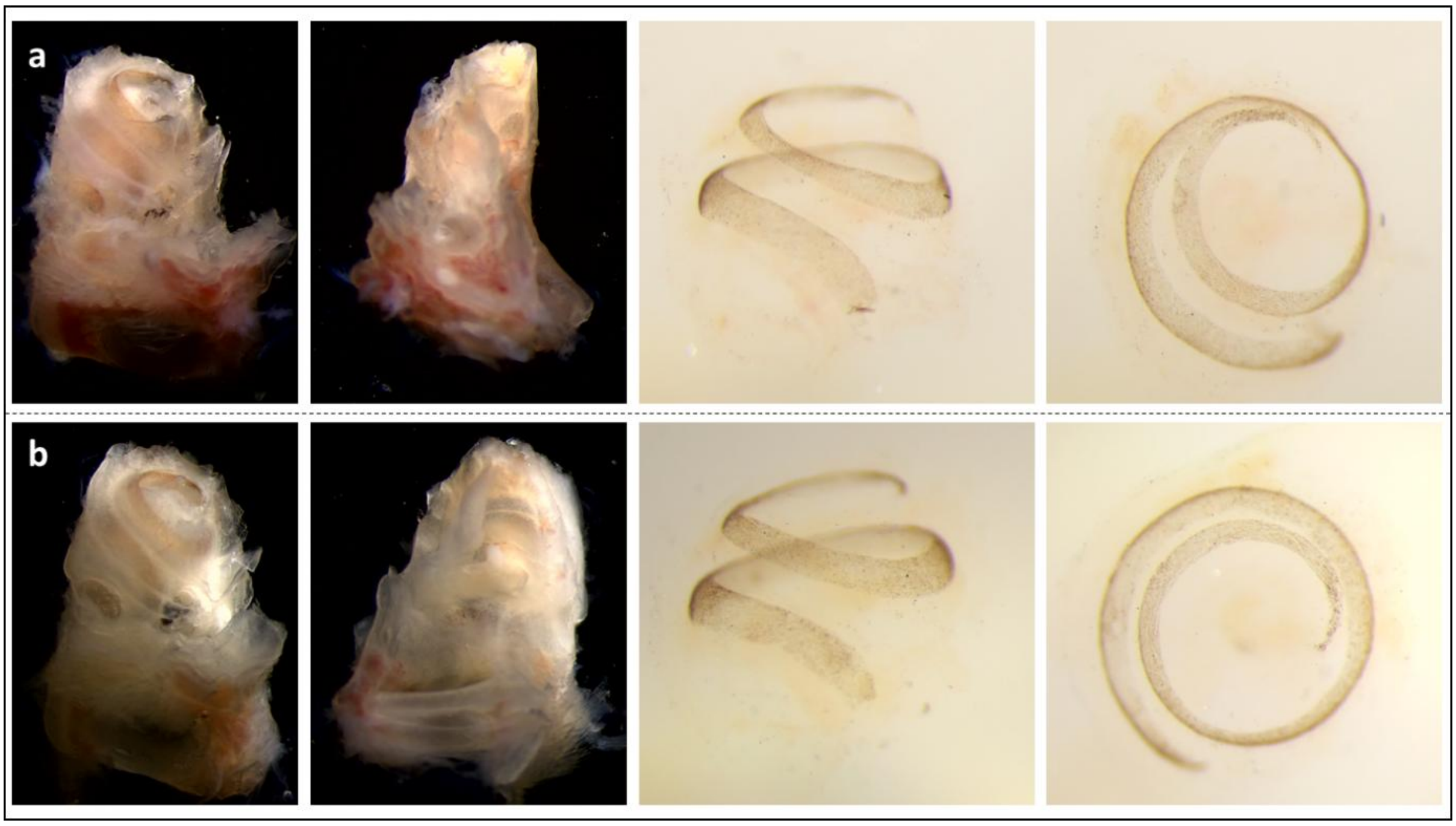

Fig. S7: Comparison of cochleae with and without transcardial perfusion. Representative outcome of a non-perfused (**a**) and perfused (b) animal, the cochleae of which were cleared with fastECi. No difference in the pigmentation level of the stria vascularis was observed. Age of animals: 9 months.

Table S1: List of antibodies used in this study.

Antibody	Host	Company	Cat No.	Dilution	Success
Calretinin	Goat	Swant	CG1	1:200	Yes
Myosin VIIa	Rabbit	Proteus Bioscienes Inc.	25-6790	1:400	Yes
Oncomodulin	Rabbit	Swant	OMG4	1:200	Yes
Parvalbumin	Guinea pig	Synaptic System	195 004	1:450	Yes
Beta III Tubulin	Rabbit	Sigma-Aldrich	T2200	1:100	Yes
Vglut3	Guinea pig	Synaptic Systems	135 204	1:400	Yes
Alexa Fluor® 647	Donkey anti-Rabbit IgG (H+L)	Jackson ImmunoResearch	711-606-152	1:800	Yes
Alexa Fluor® 790	Donkey anti-Guinea Pig IgG (H+L)	Jackson ImmunoResearch	706-655-148	1:400	Yes
Alexa Fluor™ 546	Donkey anti-Rabbit IgG (H+L)	Invitrogen	A10040	1:200	Yes
Alexa Fluor™ 594	Donkey anti-Goat IgG (H+L)	Invitrogen	A-11058	1:400	Yes
Alexa Fluor™ Plus 647	Donkey anti-Goat IgG (H+L)	Invitrogen	A32849	1:400	Yes
SYTOX™ Green nucleic acid stain		Invitrogen	S7020	1:5000	Yes

Table S2: Recommended pre-incubation time in 200 mM EDTA before extended trimming of the cochlear bone, depending on the age of the mouse.

**Age of mouse**	**Recommended pre-incubation in EDTA**
P28	30 – 60 min
3 months	1 – 2 h
6 months	2– 2.5 h
12 months	2.5 – 3 h

Table S3: Numbers of cochleae for each protocol used for evaluation

	**i/cDISCO**	**fastDisco**	**ECi**	**fastECi**	**PEGASOS**	**Sca*l*eS**	**FRUIT**
**$n_{cochleae}$**	10	17	10	20	10	8	4

Bench Protocol

fastECi for mouse cochlea

Collect samples

1. Take out the inner ear from the skull, remove pigmented bony shelf behind the apical part of the cochlea, make a hole in the cochlear apex, puncture oval and round window membrane
2. Fixation in 4% FA/PBS 3 h at 4 °C

Decalcification

1. Wash 3 x 10 min with PBS
2. (For optional extended trimming) Incubate for 0.5 - 3 h in 200 mM EDTA at 37 °C, depending on the age of your animal (Table S2)
3. (Optional) Dissect unnecessary tissue and bone, removal of semicircular canals
4. Incubate in 200 mM EDTA 24 h at 37 °C (or three days for the less trimmed cochlea)

Immunolabeling

1. Wash 3 x 10 min with DMSO
2. Wash 3 x 20 min with PBS
3. Incubation in blocking solution at 37 °C for 2 h
4. Primary antibody in blocking solution at 37 °C overnight
5. Wash 3 x 30 min in washing buffer at 37 °C
6. Secondary antibody in blocking solution at 37 °C overnight, light protected

Dehydration

1. Wash 3 x 15 min in washing buffer at 37 °C
2. Wash 2 x 15 min in PBS at 4 °C: use last washing step to dissect any remaining tissue and bone, especially macroscopic blood vessels
3. Use 1 ml syringe without tip for agarose embedding. Use 70 µl of 1% low melt agarose per cochlea
4. Dehydrate in increasing series of ethanol/ddH_2O, each for 1 h:
   20%, 40%, 60%, 80%, 100%
5. Change 100% ethanol and leave overnight

Clearing

1. Discard ethanol and change solution to ethyl cinnamate, incubate for at least one day; invert tube occasionally

Solutions and buffers for fastECi

**Solution/buffer**	**Composition**
Fixation buffer	4% FA/PBS
200 mM EDTA	Tetrasodium Tetrahydrate Salt/ddH_2O
Blocking solution	DSDB: 17% donkey serum, 0.3% Triton X-100, 20 mM phosphate buffer, 0.45 M NaCl/ ddH_2O
Washing buffer	PBS-T (1% Triton-X100)

Dehydration series	Increasing Ethanol/ddH_2O: 20%, 40%, 60%, 80%, 100%

FastDISCO for mouse cochlea

Collect samples

3. Take out the inner ear from the skull, remove pigmented bony shelf behind the apical part of the cochlea, make a hole in the cochlear apex, puncture oval and round window membrane
4. Fixation in 4% FA/PBS, 3 h at 4 °C

Decalcification

5. Wash 3 x 10 min with PBS
6. (Optional for extended removal of cochlear bone) Incubate for 0.5 - 4 h in 200 mM EDTA at 37 °C, depending on the age of the animal
7. (Optional) Dissect unnecessary tissue and bone, removal of semicircular canals
8. Incubate in 200 mM EDTA 24 h at 37 °C (or three days for the less trimmed cochlea)

Alternative pre-treatment

1. Incubate in 25% Quadrol for 2 h
2. Wash 3 x 10 min with PTx.2
3. Wash 3 x 10 min with PTD at 37 °C
4. Wash 3 x 10 min with PTTDDI at 37 °C
5. Wash 3 x 10 min with PTx.2

Immunolabeling

1. Incubation in permeabilization solution 30 min at 37 °C
2. Incubation in blocking solution 2 h at 37 °C
3. Primary antibody in blocking solution at 37 °C overnight
4. Wash 3 x 10 min in PTwH at 37 °C
5. Secondary antibody in blocking solution at 37 °C overnight, light protected

Dehydration

1. Wash 3 x 10 min in PTwH at 37 °C: use last washing step to dissect any remaining tissue and bone, especially blood vessels
2. Use 1 ml syringe without tip for agarose embedding. Use 70 µl of 1% low melt agarose per cochlea
3. Dehydrate in increasing series of ethanol/ddH_2O, each for 1 h:
   20%, 40%, 60%, 80%, 100%
4. Change 100 % ethanol and leave overnight

Clearing

1. Wash 3 h in 66% DCM/33% ethanol
2. Wash 2 x 15 min in 100% DCM
3. Discard DCM and change to DBE

Solutions and buffers for fastDISCO

**Solution/buffer**	**Composition**
Fixation buffer	4% FA in PBS
EDTA	200 mM Tetrasodium Tetrahydrate Salt in ddH_2O
Quadrol	25% N,N,N',N'-Tetrakis(2-Hydroxypropyl)ethylenediamine in PBS
PTx.2	0.2% Triton X-100 in PBS
PTD	0.2% Triton X-100/20% DMSO in PBS
PTTDDI	0.1% Tween-20/0.1% Triton X-100/0.1% Deoxycholate/0.1% IGEPAL Ca-630/20% DMSO in PBS
Permeabilization solution	2.3% Glycine/20% DMSO in PTx.2
Blocking solution	DSDB: 17% donkey serum, 0.3% Triton X-100, 20 mM phosphate buffer, 0.45 M NaCl in ddH_2O
Heparin stock	10mg/ml in ddH_2O
PTwH	0.2% Tween-20/0.1% 10 mg/ml Heparin Stock/10% 10x PBS in ddH_2O
Low-melt agarose	1% in ddH_2O
Dehydration series	Increasing Ethanol/ddH_2O: 20%, 40%, 60%, 80%, 100%
DCM	66% dichlormethane/33% ethanol
DBE	100% Dibenzylether

Trouble shooting and bench advice

- Use 2 ml tubes for collecting the cochlea and all the remaining steps. Use 1 ml of solution for washing steps, fill tube completely with clearing solution
- 100 - 150 µl of antibody solution is sufficient to cover the cochlea
- If not other mentioned, steps were performed at room temperature
- Incubation of FA can be extended to overnight; incubation of EDTA can be extended to 14 days without negative impact; incubation of antibodies can be extended to 3 days each
- A lot of clearing protocols use shaking or rotation when incubating samples, however, since we had problems with mechanical stress during our first trials, we decided to refrain from this
- be sure to use fresh dehydration solvents, since DCM is highly immiscible to water and can cause shrinkage and milky appearance of agarose if water is present; if this happens, rehydrate and dehydrate again
- longer incubation in the clearing solution only showed positive effects in DBE, which cleared all leftover pigments; this did not work with any other clearing solution (more so, FRUIT solution turned yellow and PEGASOS led to morphological alteration)
